# CPAP3 proteins in the mineralized cuticle of a decapod crustacean

**DOI:** 10.1038/s41598-018-20835-x

**Published:** 2018-02-05

**Authors:** Shai Abehsera, Shir Zaccai, Binyamin Mittelman, Lilah Glazer, Simy Weil, Isam Khalaila, Geula Davidov, Ronit Bitton, Raz Zarivach, Shihao Li, Fuhua Li, Jianhai Xiang, Rivka Manor, Eliahu D. Aflalo, Amir Sagi

**Affiliations:** 1 Department of Life Sciences, Ben-Gurion University of the Negev, Beer-Sheva, Israel; 2 The National Institute for Biotechnology in the Negev, Ben-Gurion University of the Negev, Beer-Sheva, Israel; 3 Department of Biotechnology Engineering, Ben-Gurion University of the Negev, Beer-Sheva, Israel; 4 Department of Psychiatry and Behavioral Science, Duke University Medical Center, Durham, USA; 5 Key Laboratory of Experimental Marine Biology, Institute of Oceanology, Chinese Academy of Sciences, Qingdao, China

## Abstract

The pancrustacean theory groups crustaceans and hexapods (once thought to comprise separate clades within the Arthropoda) into a single clade. A key feature common to all pancrustaceans is their chitinous exoskeleton, with a major contribution by cuticular proteins. Among these, are the CPAP3’s, a family of cuticular proteins, first identified in the hexapod *Drosophila melanogaster* and characterized by an N-terminal signaling peptide and three chitin-binding domains. In this study, CPAP3 proteins were mined from a transcriptomic library of a decapod crustacean, the crayfish *Cherax quadricarinatus*. Phylogenetic analysis of other CPAP3 proteins from hexapods and other crustaceans showed a high degree of conservation. Characterization of the crayfish proteins, designated CqCPAP3’s, suggested a major role for CPAP3’sin cuticle formation. Loss-of-function experiments using RNAi supported such a notion by demonstrating crucial roles for several CqCPAP3 proteins during molting. A putative mode of action for the CqCPAP3 proteins –theoretically binding three chitin strands– was suggested by the structural data obtained from a representative recombinant CqCPAP3. The similarities between the CqCPAP3 proteins and their hexapod homologues further demonstrated common genetic and proteinaceous features of cuticle formation in pancrustaceans, thereby reinforcing the linkage between these two highly important phylogenetic groups.

## Introduction

The Pancrustacean theory groups together into a single clade the Crustacea and the Hexapoda, two taxa that were once thought to be separate clades within the Arthropoda^1–3^. One of the defining characteristics common to all pancrustaceans and arthropods in general, is the presence of a rigid exoskeleton whose principal organic components are chitin and proteins. The rigidity of the exoskeleton dictates that all arthropods must undergo a molt cycle if they are to metamorphose and grow. Thus, many of the features of the exoskeleton composition and of the molt cycle are common across the ‘modern’ pancrustacean taxa and presumably predate the divergence of the Crustacea and the Hexapoda^4^.

In the Crustacea – the taxon of interest to us here – the molt cycle is composed of four main consecutive stages: inter-molt, the stage between two molting events during which the animal has a fully developed exoskeleton; pre-molt, during which the old exoskeleton is partly reabsorbed in parallel to the initial formation of the new exoskeleton; ecdysis, the actual act of shedding the old exoskeleton; and post-molt, during which the cuticle continues to be deposited and is hardened through sclerotization and mineralization^5^.

Our study animal, the decapod crustacean *Cherax quadricarinatus* (Cq), is characterized by two cuticular structures – the gastrolith and the molar tooth – in addition to the cuticular exoskeleton, common to all crustaceans. The two gastroliths, which are located one on each side of the stomach wall, are transient calcium storage organs having a chitinous scaffold which is mineralized with stabilized amorphous calcium carbonate during the pre-molt stage (as opposed to most of the cuticle, which is mineralized with calcium carbonate in the post-molt stage). During post-molt, the gastroliths collapse into the stomach where they are fully digested, thereby providing the calcium for cuticular mineralization^6–8^. The two molar teeth are part of the mandibles of *Cherax quadricarinatus*. The mandibles form the anterior mouthparts and are located directly in front of the oral opening. The molar teeth serve as a grinding surface for mastication and processing of food. The molar teeth are cuticular structures that are mineralized with a calcium carbonate (innermost) to calcium phosphate (outermost) gradient. Unlike the rest of the cuticle but similar to the gastroliths, the enamel-like molar crown is mineralized during pre-molt^9^. The peculiar molt mineralization patterns of the gastrolith and the molar tooth – both cuticular structures – were exploited to construct the transcriptomic library^10^ that was used for the present study.

In arthropods, the formation of cuticular structures is controlled by an organic matrix in which proteins play a major role^4,11^, as is the case for most bio-materials. Numerous proteins have thus far been shown to be involved in pancrustacean exoskeleton formation, either as participants in chitin metabolism or as cuticular structural proteins^12–18^. On the basis of the relationship between the expression pattern of these proteins and molt cycle events^19–21^, we previously developed a binary patterning approach^10^ as a specialized tool for mining such cuticular proteins. In the current study, this tool was applied to reveal crustacean proteins of the CPAP3 family.

The CPAP3 family of cuticular proteins was first identified in the fruit fly *Drosophila melanogaster* in which they were named “Obstructor proteins”^22^. These proteins are characterized by a stereotypical arrangement comprising an N-terminal signaling peptide and three cysteine-rich chitin-binding domains (CBDs)^22^. One such CPAP3 protein, found in *Drosophila melanogaster* and named ObstructorA, was found to be involved in cuticle maturation by coordinating the extracellular matrix trafficking and localization of proteins and enzymes in the newly deposited cuticle of *Drosophila melanogaster*^23,24^. Later in order to further clarify the naming of this family, based on the existence of three CBD’s which are also named peritrophinA, the name of the Obstructors was modified to Cuticular Proteins Analogous to Peritrophins 3 (CPAP3) by Jasrapuria *et al*.^25^, this naming is thus utilized throughout our article. CPAP3 proteins have also been found in other insects, such as the red flour beetle *Tribolium castaneum*, in which they are also involved in cuticle formation^25,26^. But thus far albeit the fact that they were found in the genome of *Daphnia Pulex*^27,28^, they have not been characterized in any crustacean.

In the present study, we characterize for the first time the CPAP3family in a crustacean species. Characterization of these proteins revealed their phylogeny within the pancrustacea and showed similarities between prominent hexapods and the crustacean *Cherax quadricarinatus* with regard to the role of these proteins in cuticle formation during the molt cycle. Moreover, functional genomics through RNAi confirms such a role, showing that CPAP3 proteins are vital during molting having a role in the formation of the chitinous scaffold. Thus, this study demonstrates common pathways for the formation of the typical cuticular chitinous scaffold within the important phylogenetic group of pancrustacea.

## Results

### Cherax quadricarinatus CPAP3 proteins

**Figure 1:**
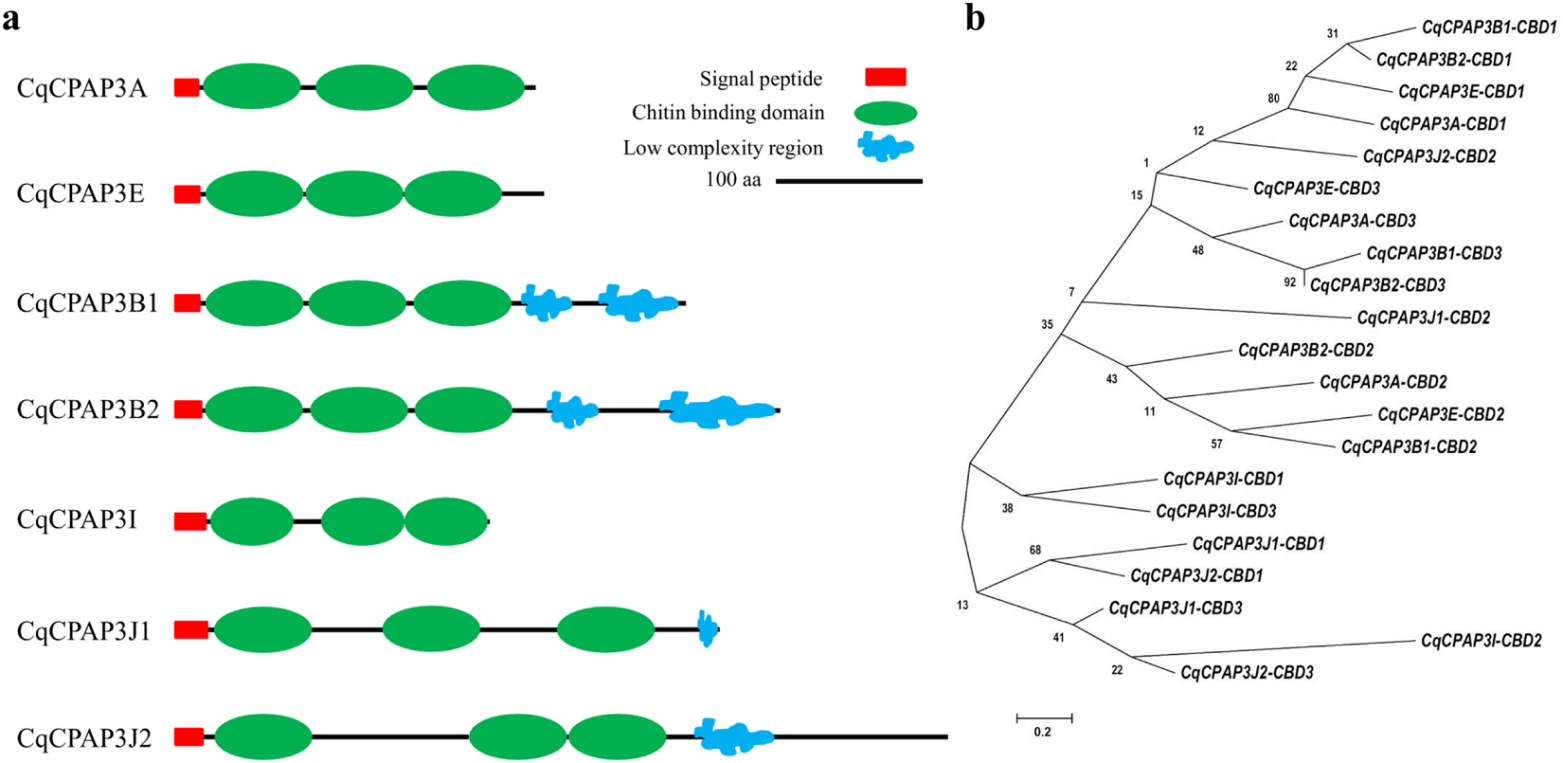
CPAP3 proteins found in the decapod crustacean *Cherax quadricarinatus* (Cq). (**a**) Schematic representation of the protein sequence of the different CqCPAP3 proteins; (**b**) Phylogeny of the chitin-binding domains (CBDs) of the CqCPAP3 proteins. Bootstrap test (n = 1000) results are shown on each node junction.

A total of seven CPAP3 proteins were identified in the molt-related transcriptomic library of *Cherax quadricarinatus* (all sequence data is presented in the supplementary material); these proteins were designated according to their phylogenetic relationship with known CPAP’s and similarities in the domain organization: CqCPAP3A (MF407543), CqCPAP3E (MF407544), CqCPAP3B1 (MF407545), CqCPAP3B2 (MF407546), CqCPAP3I (MF407547), CqCPAP3J1 (MF407548) and CqCPAP3J2 (MF407549), as shown in Fig. 1a. *In silico* characterization of these CqCPAP proteins demonstrated that, in addition to CBDs, CqCPAP3B1, B2, J1 and J2 possess a low complexity region (Fig. 1a) that is predicted to be disordered (Fig. S1). Phylogeny analysis of the different CBDs within the CqCPAP3 family (Fig. 1b) showed that CBDs 1–3 of CqCPAP3, E, B1 and B2 cluster together. Within this large cluster each CBD clusters with its similar CBDs, i.e. CBD1 from A clusters with CBD1 of E, B1 and B2, forming three sub-clusters. CBDs1-3 of CqCPAP3I, J1 and J2 also cluster together, with the exception that the CBD2s of CqCPAP3J2 and J1 cluster with the CBDs of CqCPAP3A, E, B1 and B2. For CqCPAP3I, J1 and J2, the different CBDs do not cluster in a CBD-specific cluster.

### Phylogenetic analysis

**Figure 2:**
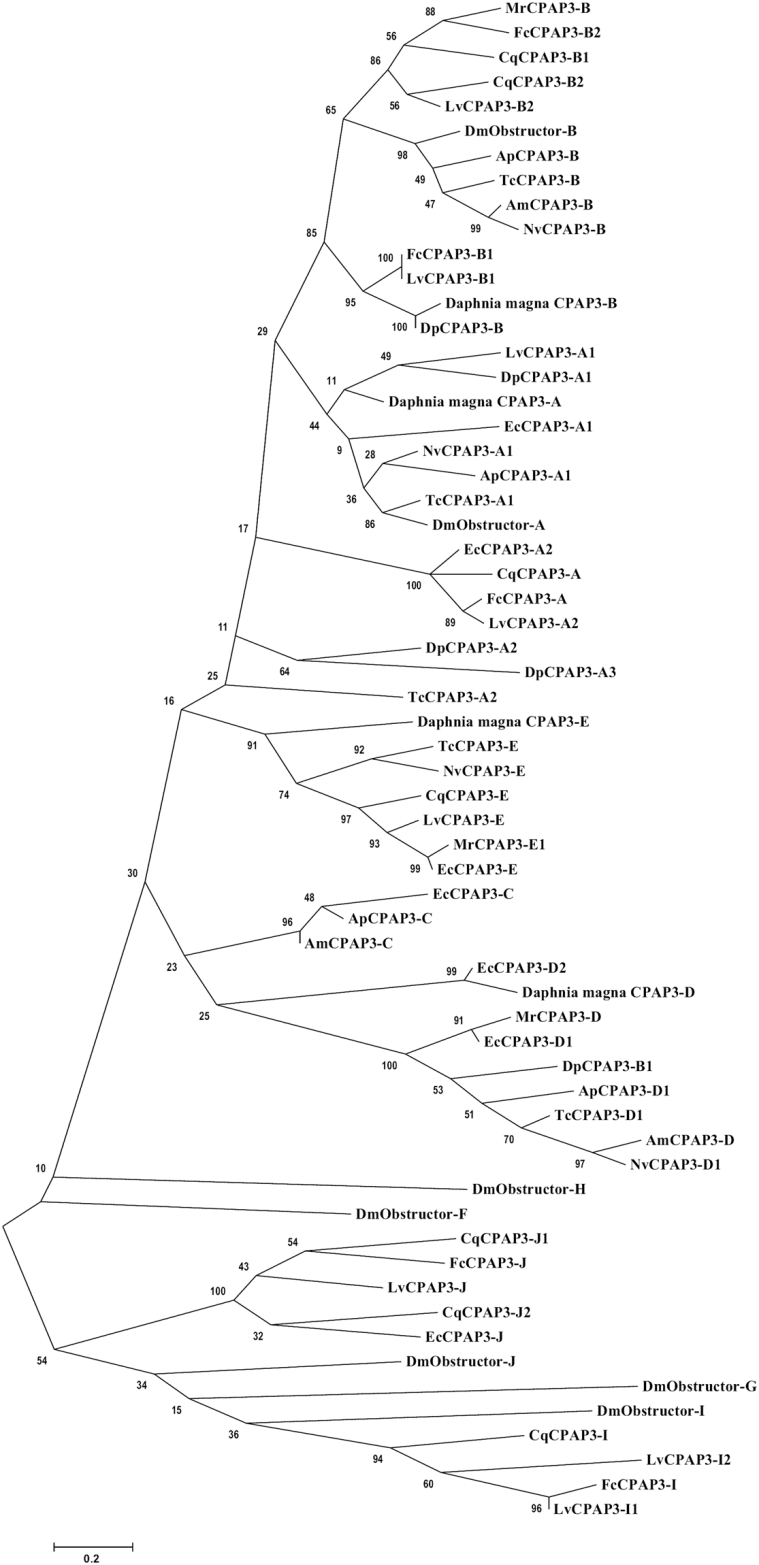
CPAP3 proteins are highly conserved within the pancrustaceans. Maximum likelihood tree of CPAP3 proteins (original tree) from several pancrustaceans, including: the decapod crustaceans *Cherax quadricarinatus* (Cq), *Palaemon carinicauda* (Pc), *Exopalamon carincauda* (Ec), *Fenneropenaeus chinensis* (Fc), *Litopenaeus vannamei* (Lv) and *Macrobrachium rosenbergii* (Mc); the branchiopod crustaceans *Daphnia pulex* (Dp) and *Daphnia magna*; and the hexapod insects *Drosophila melanogaster* (Dm)*, Tribolium castaneum* (Tc), *Nasonia vitripennis* (Nv), *Acyrthosiphon pisum* (Ap) and *Apis mellifera* (Ap). Bootstrap test (n = 1000) results are shown on each node junction.

The CPAP3 proteins from the decapod crustacean *Cherax quadricarinatus* and other crustaceans bear high homology with the CPAP3 proteins from hexapods (Fig. 2). CqCPAP3A, E, B1 and B2 cluster together in a large cluster of several homologous CPAP3 proteins from insects and different crustaceans. In this large cluster, CqCPAP3A does not cluster with other CPAP3 proteins from hexapods but it is located in the phylogenetic tree with CPAP3A proteins from other crustaceans. This crustacean specific cluster is most closely related to a cluster composed of CPAP3A proteins from insects and other crustaceans such as *Daphnia pulex*. Additionally, the domain organization of CqCPAP3A indicates that this CPAP3 is homologues to CPAP3A of insects. CqCPAP3B1 and B2 cluster together in a larger cluster that includes CPAP3B from *Drosophila melanogaster*, *T. castaneum* and *Apis mellifera* all having a similar domain organization including a low complexity region at the C terminal. Additionally, they cluster with CPAP3 (also designated CPAP3B) from different crustaceans, such as the decapod crustacean *Litopenaeus vannamei* and the branchiopod crustacean *Daphnia magna*. CqCPAP3I, J1 and J2 cluster together in a larger cluster of several homologous CPAP3 proteins from insects and different crustaceans. In this large cluster, CqCPAP3I clusters with CPAP3I proteins from different crustaceans with their closest insect homologue being CPAP3I from *Drosophila melanogaster*. Additionally, in this cluster CPAP3J from *Drosophila melanogaster* is close to two CqCPAP3 termed CqCPAP3J1 and J2, all having a similar domain organization composed of larger linkers and a C-terminal extension. In addition, they cluster with newly found CPAP3J from different crustaceans, such as the decapod crustaceans *Fenneropenaeus chinensis* and *Exopalamon carincauda*. CPAP3 proteins which were not found in our molt-related transcriptomic library of the crayfish *Cherax quadricarinatus* but are known to exist in different hexapods, such as CPAP3C and D, that were found in other crustaceans. An example is CPAP3D, which was found in the marine shrimp *Exopalamon carincauda* and in the ridgetail prawn *Palaemon carinicauda*.

### Spatial and temporal expression

Spatial and temporal expression experiments were performed to characterize the expression pattern of the *CqCPAP3* genes in different cuticular-forming epithelia during the molt cycle (Fig. S2). Two approaches were employed both an *in-silico* approach using our molt-related transcriptomic library^10^ and an *in vitro* approach using spatial temporal PCR. In both the qualitative *in vitro* (Fig. S2a) and the quantitative *in silico* from our molt-related transcriptomic library(Fig. S2b) spatial-temporal expression experiments, *CqCPAP3A*, *E*, *B1* and *B2* transcripts were found to be the main actively expressed *CqCPAP3* in the different cuticle-forming epithelia of *Cherax quadricarinatus*. For *CqCPAP3A* and *E*, *in vitro* spatial and temporal expression patterns were similar in the different tissues, with expression being evident in the different cuticular-forming epithelia in all four molt stages and in all the tested non-cuticular tissues. For *CqCPAP3B1* and *B2*, *in vitro* spatial and temporal expression was highly specific in all the different cuticular-forming epithelia and in all molt stages. With the exception of its expression in the gastrolith-forming epithelium at the inter-molt stage, *CqCPAP3I* was expressed uniquely in the hepatopancreas throughout the entire molt cycle. *CqCPAP3J1* was expressed mainly in the gastrolith-forming epithelium throughout the entire molt cycle, but expression of *CqCPAP3J2* was limited to early pre-molt in the carapace cuticle and the molar-forming epithelium (Fig. S2a). The *in silico* expression results for the molar-forming epithelium showed that the expression of *CqCPAP3A*, *B1* and *B2* was independent of the molt cycle, being high throughout the entire molt cycle, while *CqCPAP3E* had a pre-molt-related pattern of expression, with the expression being higher at both early and late pre-molt compared to that in the post- and inter-molt stages. *CqCPAP3I*, *J1 and J2* were not highly expressed in the molar-forming epithelium, giving only a small number of reads or none at all throughout the molt cycle (Fig. S2b left). The *in silico* expression experiments for the gastrolith-forming epithelium showed that *CqCPAP3A*, *E*, *B1*, *B2* and *J1* had molt-related patterns of expression: In particular, high expression was observed for *CqCPAP3A* and *B2* at early premolt and post molt, for *CqCPAP3E* at post-molt, and for *CqCPAP3B1* at pre-molt. *CqCPAP3I* was found to have no expression in a cuticular tissue in the *in vitro* experiments while *CqCPAP3J2* was found to have a specific expression in the *in vitro* experiments. Both gave only a small number of reads or none at all throughout the molt cycle (Fig. S2b right).

### Analysis of cuticular proteins

To characterize the CqCPAP3 proteins in the cuticular structures of the mature crayfish, three steps of solvent extraction were performed, followed by dialysis yielding three fractions (EGTA soluble, urea soluble and guanidinium thiocyanate soluble) that were then separated by SDS-PAGE. Thereafter, proteins were analyzed by tandem mass spectrometry. Presence of CqCPAP3 proteins was validated using MS\MS and it was found that they were recovered from the different cuticular structures (Fig. S3). Only CqCPAP3A was identified and validated using MS\MS in the 30-kDa band in most of the fractions, with the exception of the guanidinium-soluble fraction. Only CqCPAP3E was identified and validated using MS\MS in the 32-kDa band of the guanidinium-soluble fraction from the cuticle. Only CqCPAP3B1 was identified and validated using MS\MS in a 39-kDa band only in the EGTA-soluble fraction from the gastrolith. Only CqCPAP3B2 was identified and validated using MS\MS in the 50-kDa band of the urea-soluble fraction from the molar tooth. CqCPAP3I, J1 and J2 were not detected in the different fractions.

### Phenotypic effects of RNAi

**Figure 3:**
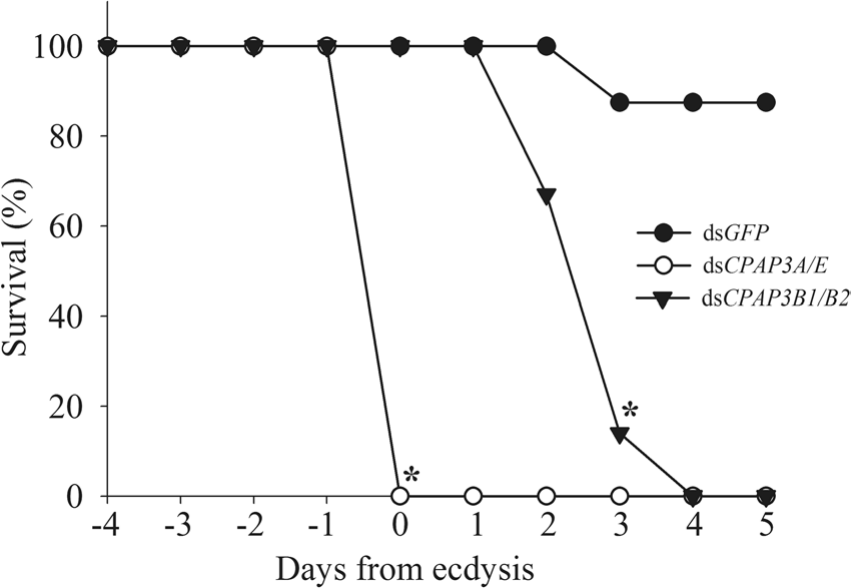
The CqCPAP3 proteins are vital during the molt cycle of *Cherax quadricarinatus*. Graph showing survival rates in the ‘post-molt experiment’. The control group was injected with ds*GFP* (n = 8); the treatment groups were injected with either ds*CqCPAP3A*/*E* (n = 7) or ds*CqCPAP3B1*/*B2* (n = 7. All groups were given ecdysone injections to induce molting. Asterisk indicates the first day with a significant difference in survival rate of a treatment group compared to the control group (*P* < *0.05*).

**Figure 4:**
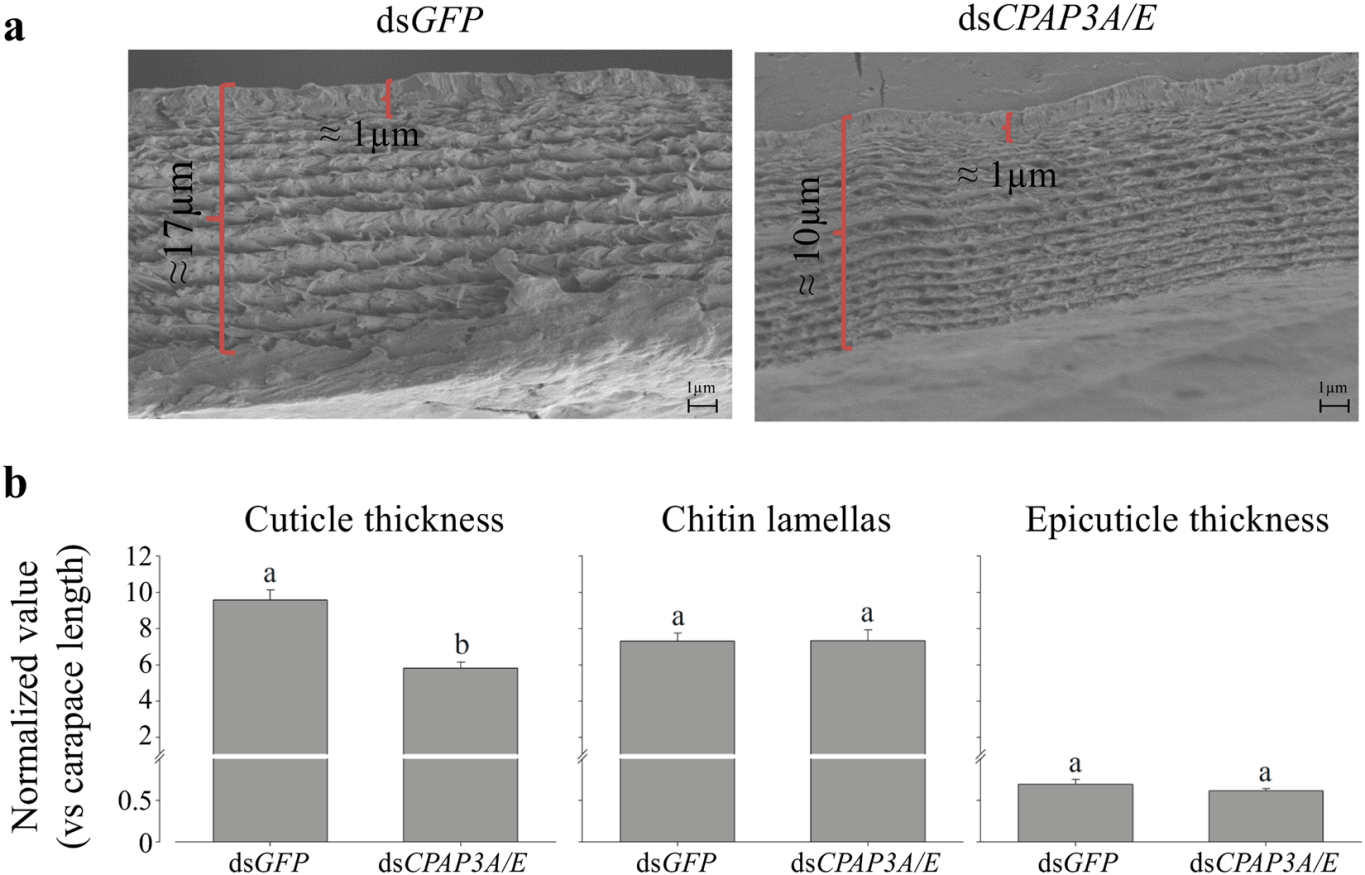
CqCPAP3A and E are important for correct cuticle development at pre-molt. (**a**) Representative newly formed cuticles of similar size animals at the onset of ecdysis. The thickness of the cuticle and epicuticle are indicated on the photograph. (**b**) Normalized values (normalized against the carapace length) of cuticle thickness, number of chitin lamellas and epicuticle thickness of the carapace cuticle of animals injected with ds*GFP* (n = 8) compared to animals injected with ds*CqCPAP3A/E* (n = 9). Different letters represent statistical groups that are significantly different (p-value < 0.05); error bars represent standard error.

To elucidate the role of the CPAP3 proteins in the molt cycle, two loss-of-function experiments were conducted in parallel to molt cycle induction, namely, a ‘post-molt experiment’ (Fig. 3) in which *CPAP3* transcripts were silenced throughout pre- and post-molt periods to test for potential post-molt lethality, and a ‘pre-molt experiment’ (Fig. 4) in which *CPAP3* transcripts were silenced only during the pre-molt period to identify structural effects of the gene silencing on new cuticle formation. The ‘post-molt experiment’ showed that co-silencing *CqCPAP3A/E*, or *CqCPAP3B1*/*B2* induces lethality demonstrating that they are vital during the molt cycle (Fig. 3), with a different mortality pattern being seen in each treatment group. *CqCPAP3*A- and E-silenced groups showed a drop to 0% survival on the day of ecdysis, with a significant reduction in survival being evident on that day. *CqCPAP3*B1- and B2-silenced groups showed a significant drop to 14% survival on the day of ecdysis +3 and a decline to 0% survival one day thereafter. The control group exhibited a high survival rate of 87% to the end of the experiment.

The ‘pre-molt experiment’ showed that CqCPAP3A and E both play a role in the formation of the cuticle at pre-molt (Fig. 4). The cuticle of the silenced group showed a significant reduction in thickness (≈40%, as measured and normalized against the carapace length) vs the control group (Fig. 4a and b, left graph). The number of chitin lamellas and the epicuticle thickness, which were also measured and normalized against the carapace length, were similar in the control and silenced groups (Fig. 4b, middle and right graphs). Silencing efficiency was tested using qPCR; a significant reduction in the expression of *CqCPAP3*A, E, B1 and B2 in the silenced group compared to the control group gave a silencing efficiency of ≈95% (Fig. S4).

### Characterization of rCqCPAP3A

**Figure 5:**
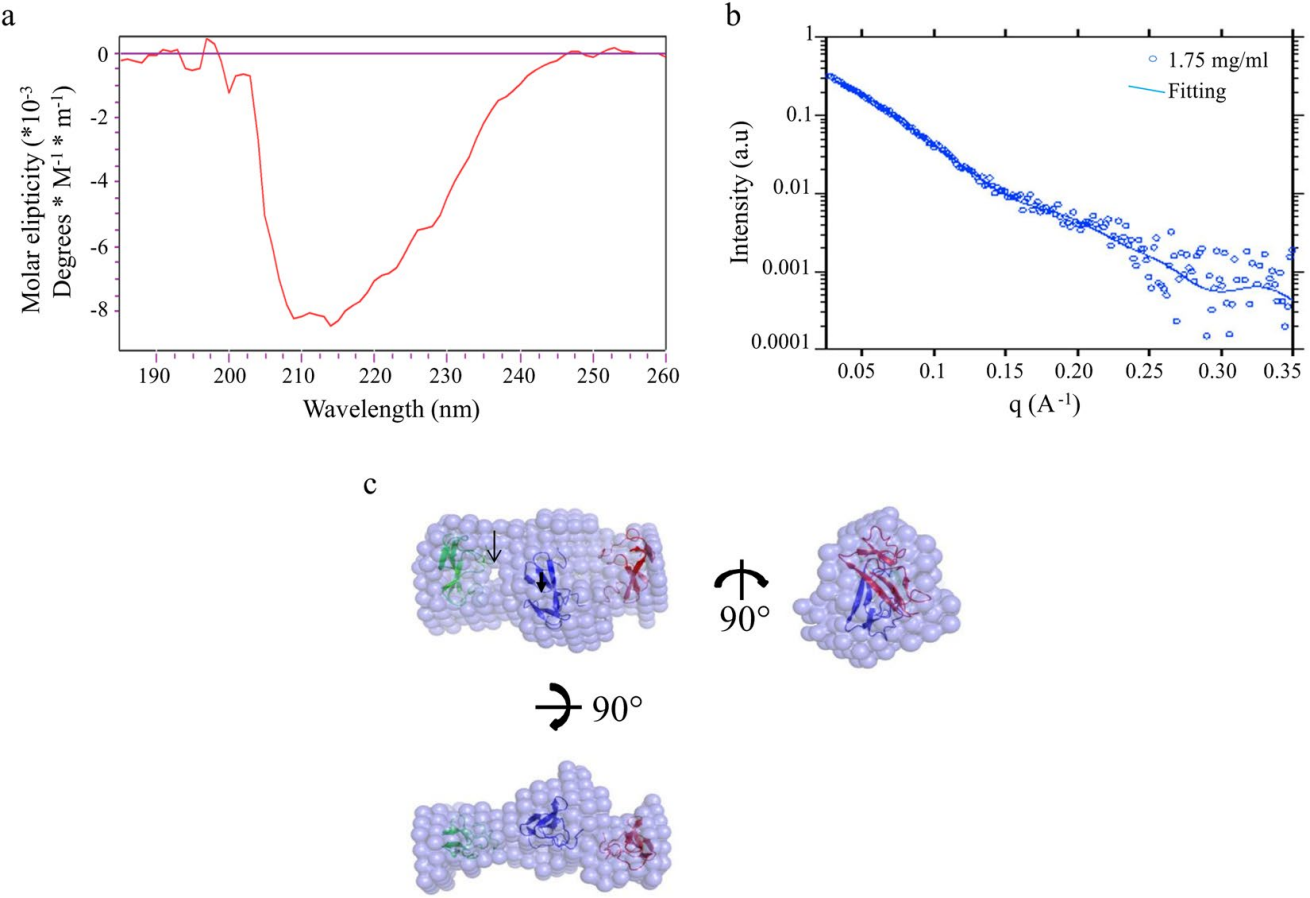
rCqCPAP3A is shown to have a typical three CBD-containing protein structure. (**a**) CD spectra of rCqCPAP3A. (**b**) SAXS analysis of rCqCPAP3A (dots represent experimental data, and line shows fit). (**c**) Dummy-ball model (DBM) of 1.75 mg/ml (70 μM) fitted onto three copies of the core deposited structure of Tachycitin (PDB code 1DQC). A gap in the dummy-ball map is marked with a black arrow.

To elucidate the mechanism of function of the typical CPAP3 protein, we chose rCqCPAP3A as a representative of the family in *Cherax quadricarinatus* for characterization based on the production of a recombinant rCqCPAP3A protein. This choice was made based on its abundance in all the cuticular structures and its relatively simple domain arrangement. The protein was successfully purified from *E*. *coli* as a monomer in solution. The rCqCPAP3A was validated using MS/MS of 15 unique peptides covering 93.7% of the protein sequence, with a sequest score of 754.8 (data not shown). The secondary structure of rCqCPAP3A was studied by CD spectrometry (Fig. 5a) and the 3D structure was studied via SAXS (Fig. 5b). Secondary structure distribution determined by CD-spectrometry between 190–260 nm was 6% for α-helices, 49% for β-strands, 16% for β-turns and 29% for random coils. The monomeric state together with the protein β-sheet fold indicates that the protein was folded with the correct disulfides which otherwise would lead to protein aggregation. The 1D SAXS profile served as a platform to generate a low resolution dummy-ball models (DBMs) of rCqCPAP3A through the use of DAMMIN^29^. As anticipated, rCqCPAP3A exhibited an elongated cylindrical shape (seen from three angles in Fig. 5c), which could be back fitted to the raw SAXS data (Fig. 5b). Tachycytin resolved ChtBD2 that matches rCqCPAP3A domain via its distribution of cysteines and a 3 disulfides^30^ was used as a model representation for each of the rCqCPAP3A domains. Three copies of the Tachycytin ChtBD2 were superimposed on the experimentally generated DBM, while maintaining the proper N′-terminal to C’-terminal alignment. The domains were positioned vertically to each other, with a distinguishable gap in the dummy atom map between the first and second domains, as seen in Fig. 5c. However, it is not known how the sequences connecting the three domains are spatially arranged.

## Discussion

In this study, CPAP3 proteins are characterized in a crustacean. The use of a molt-related transcriptomic library and a binary patterning approach^10^ facilitated the identification of several CPAP3 family members in the crayfish *Cherax quadricarinatus*. Further mining in other crustacean transcriptomic libraries^27,31,32^ revealed that CPAP3 family proteins are found not only in the crayfish but also in several crustaceans such as *Palaemon carinicauda*, *Exopalamon carincauda* and *Daphnia pulex*. The conservation showing a similar grouping of CPAP3 proteins in *Cherax quadricarinatus*, other crustaceans and insects within the pancrustacea supports an evolutionary link between the Hexapoda and the Crustacea. CPAP3 proteins are also found in other arthropods such as chelicerates as can be found in the gene bank, but no study on the involvement of the CPAP3 family in chelicerates or other arthropods besides insects is found in the literature. Proteins homologous to most of the known CPAP3 proteins in insects were found either in *Cherax quadricarinatus* or in other crustaceans. Those hexapod CPAP3 proteins for which no crustacean homologues were found might have evolved after the separation of hexapods from crustaceans or were simply not revealed due to technical limitations of our methodology and the lack of an available *Cherax quadricarinatus* genome. As has been suggested for *Drosophila melanogaster*^22^, the CPAP3 proteins found in *Cherax quadricarinatus* could be phylogenetically divided into two subgroups, the first subgroup consisting of CqCPAP3A, E, B1 and B2 and the second, of CqCPAP3I, J1 and J2. The second group of CPAP3’s was so far found only in the fruit fly *Drosophila melanogaster*^22^. Since the second group of CPAP3 proteins was so far found only in *Drosophila melanogaster*, all the homologues of these proteins are named Obstructor as suggested by Behr & Hoch^22^. In our study we have decided to name them CPAP3 proteins according to the new nomenclature as suggested by Jasrapuria *et al*.^25^ CPAP3 proteins. This decision was made since these proteins share similar characteristics (three perithropinA domains) and for reasons of uniformity and clarity. The fact that these CPAP3’s were also found in several crustaceans is supporting their possible occurrence across the pancrustacea. The low bootstrap test results seen in the maximum likelihood trees present a challenge but contributes to our understanding of this gene family. Moreover, such difficulties in the phylogenetic analysis seem to be related to the simple domain organization of the CPAP3 proteins all having similarly three consecutive chitin binding domains with, for most of the CPAP3’s, short linkers. Additionaly Such a phylogenetically based grouping seems to be related to the function of the CPAP3 proteins. Spatial-temporal expression and protein purification results indicated that in the crayfish the first – most abundant – subgroup contains the major proteins in the different cuticular structures, while the second subgroup seems to include specialized CPAP3 proteins that are expressed in a specific manner. A putative role for specialized CPAP3’s could be deduced from their spatial expression. For example *CqCPAP3I*, expressing mainly in the hepatopancreas, a non cuticular tissue known to be involved in the metabolism of chitin^33^ might be related to this function. *CqCPAP3J1* and J2 which are temporally expressed during the molt cycle in cuticular tissues might suggest an involvement during specific molt events. For example, the formation of the gastroliths for CqCPAP3J1 or the formation of the exocuticle at premolt for CqCPAP3J2.

To date, the roles of CPAP3 family proteins have been studied only in insects, in which they were found to be involved in cuticular structure formation based on their ability to bind to the chitinous scaffold^22–24,26^. Similarly, in the crustacean species, *Cherax quadricarinatus*, loss-of-function experiments using RNAi confirmed the role of CqCPAP3 proteins in cuticle formation during the molt cycle. This notion is supported by the mortality in the ‘post-molt experiment’. Bearing in mind that silencing – engineered by several weeks of dsRNA injections prior to molting – did not affect survival showing that the CPAP3’s role is not related to vital functions that are not related to molt. Thus, the role of the CPAP3’s in *Cherax quadricarinatus* seems to be intimately linked to specific molt cycle events. The finding that when CqCPAP3A and E were knocked down the animals could not survive ecdysis indicates that a reduction in the content of these CqCPAP3’s causes cuticular defects that impair the protective core function of the cuticle. The drop in survival seen during post-molt following the knock down of CqCPAP3B1 and B2 is probably related to molt cycle events occurring at post-molt, such as the maturation or mineralization of the cuticle^5^. The silencing experiments were conducted by co-silencing of two CPAP3’s since in preliminary experiments aiming at one CqCPAP3 at a time no phenotypic results were found. A compensation mechanism between similar CqCPAP3’s was hypothesized. The choice of which CqCPAP3’s are to be silenced together was made based on phylogenetic relationships. In *Drosophila melanogaster*^23^ knocking down of ObstructorA resulted with impaired larval growth and survival ^24^and in *T*. *castaneum* CPAP3 proteins were found to be important for adult survival^25^.In addition, in those insects loss-of-function experiments for the CPAP3’s triggered deformities in cuticular structures^23–25^. Similar to the cuticular deformities seen in the insects, those found in our ‘pre-molt experiment’ in a crustacean demonstrated the involvement of the CPAP3’s in the formation of cuticular structures. These deformities included a reduction in the thickness of the late pre-molt newly forming cuticle, which may be attributed to a denser stacking of the chitin lamellas. This suggestion is based on the finding that there was no reduction in either the epicuticle thickness or number of lamellas. These results attribute a role for CqCPAP3A and E in the formation of the three-dimensional structure of the chitinous scaffold.

The cuticular chitinous scaffold of crustaceans consists of a chitin-protein complex with a typical twisted plywood arrangement^34^, in which the chitin–protein matrix structure has a network-type arrangement with parallel and branched fibers. It has been suggested that the branches have an inner structure that is made up of a three-dimensional protein network^35^. On the basis of studies in insects and the results of the current study, it appears that CqCPAP3A and E are components of such an inner chitin-protein three-dimensional structure. A thorough study of the role of ObstructorA in *Drosophila melanogaster*^23,24^ showed that is protein is involved in the maturation and stabilization of the apical extracellular matrix of the insect’s cuticle. In particular, ObstructorA was found to coordinate with three other proteins, Serpentine, Vermiform and Knickkopf, to form a core protein complex with chitin^23,24^. Serpentine and Vermiform are conserved deacetylase domain-containing proteins, which are needed to enhance chitin matrix maturation, stability, and structural durability^36,37^, while Knickkopf organizes chitin and protects it from chitinase degradation at pre-molt^38^. Such protein complexes have indeed been found in the gastroliths of *Cherax quadricarinatus*, with the core protein being GAP65 – a protein that is homologous to a Serpentine/Vermiform deacetylase domain-containing protein^12,18^. Knickkopf, too, has been found in the gastroliths and cuticle of *Cherax quadricarinatus*^4^. The existence of these cuticular protein complexes in *Cherax quadricarinatus* together with the conservation of CqCPAP3A suggest that the role of CqCPAP3A might be similar to the complex-forming role of ObstructorA in *Drosophila melanogaster*. Similarly, other CqCPAP3 proteins might also form protein complexes, as is indicated by the presence of predicted intrinsically disordered regions (IDR) in CqCPAP3B1, B2, J1 and J2. In other IDR-bearing proteins, the structure of the IDRs is achieved only upon binding to a substrate in a disorder-to-order transition^39,40^. In the extracellular matrix of the crayfish, such a substrate is most likely to be a structural protein, such as GAP65, which is known to form protein complexes as described above^12,18^. The possible involvement of CqCPAP3A in the formation of this presumed three-dimensional structure is further supported by the deduced structure of the recombinant CqCPAP3A. Having such a domain arrangement (which was obtained by superimposing three copies of Tachycitin on the elongated cylindrical shape) facilitates multidirectional binding of three chitin strands. This putative structure suggests that CqCPAP3A may link several individual chitin strands into a complex structure, utilizing a binding mechanism similar to that of Tachycitin^41^. Thus, we suggest that CqCPAP3A acts as a linker in the branches of the three-dimensional network of the chitin-protein scaffold. In addition, as found in *Drosophila melanogaster*, CqCPAP3A might act as a linker between these chitinous scaffold branching points and different proteins^23,24^. Since CqCPAP3seems to be the most prominent CqCPAP3 protein in our study crustacean, as was previously found in insects^23,24^, its putative role as a three dimensional linker seems to be of major importance in the formation of the pancrustacean cuticle.

The discovery and characterization of CPAP3 proteins in a crustacean and the first demonstration that these proteins share a common role in an insect and a crustacean in cuticle formation indicate common pathways of cuticle formation within the pancrustacea. The role of CPAP3 proteins seems related to their predicted ability to bind three chitin strands, thus forming the three-dimensional structure of the protein-chitin cuticular scaffold. The results of this study thus provide valuable insights into the evolution and molecular mechanisms underlying the formation of cuticular structures in the pancrustacea, thereby reinforcing the link between members of this important phylogenetic group.

## Materials and Methods

### Animal and molt induction

*Cherax quadricarinatus* crayfish were grown in artificial ponds at Ben-Gurion University of the Negev (BGU), Beer-Sheva, Israel. Food comprising shrimp pellets (Rangen Inc., Buhl, ID, USA, 30% protein) was supplied *ad libitum* three times a week. The temperature was kept at 27 ± 2 °C, and a photoperiod of 14 h light and 10 h dark was applied. Water quality was assured by circulating the entire volume of water through a bio-filter. The pH of the water was 8.3 ± 0.5, the nitrite concentration was less than 0.1 mg/L, the nitrate concentration was less than 50 mg/L, ammonium levels were negligible, and oxygen levels exceeded 5 mg/L. For all molt induction experiments, inter-molt crayfish were held in individual cages and endocrinologically induced to enter pre-molt through daily α-ecdysone injections, as previously described^42^. Progression of the molt cycle was monitored daily by measuring the gastrolith using a non-invasive X-ray as previously described in Shechter *et al*.^43^ and the molt mineralization index (MMI), which correlates with molt stages and hormonal titers^43,44^ was calculated. MMI values for the molt stages were: inter-molt, 0; early pre-molt, 0.02–0.04; and late pre-molt 0.1–0.2. Post-molt animals were harvested on the day following ecdysis. For all dissection procedures, crayfish were placed on ice for 10–15 min until they became anesthetized.

### *In-silico* mining for CPAP3 proteins

Mining for CPAP3 proteins in *Cherax quadricarinatus* was conducted using a molt-related transcriptomic library^10^. In brief, a reference *Cherax quadricarinatus* transcriptome was constructed from next generation sequencing (NGS) of samples originating from two types of epithelial structure, the molar tooth and the gastrolith. Animals in four distinct molt stages were sampled for each type of epithelium: inter-molt (one pool of three animals, i.e., n = 1), early pre-molt (one pool of three animals, i.e., n = 1), late pre-molt (two single animals and one pool of two animals, i.e., n = 3) and post-molt (two single animals, i.e., n = 2). Normalization of the raw read counts to account for library size and differential expression analysis was performed using the DESeq R package^45^. Filtering for candidate mandible-forming proteins was performed using the binary-patterning mining approach described in a previous study^10^. Candidate *CqCPAP3*-coding genes were first mined using the binary patterning approach based on lists of contigs having various molt-related binary patterns^10^. Proteins having the characteristics of the CPAP3 protein family^22^, i.e., a signal peptide followed by three CBDs, were selected as putative CqCPAP3 proteins. Additional CqCPAP3 protein transcripts were searched using BLAST^46^ against the molt-related transcriptomic library of the newly found CPAP3’s. The presence of three consecutive CBDs (IPR002557) and other domains and regions within the newly found CPAP3 proteins from *Cherax quadricarinatus* were searched using SMART^47^. The presence of a signal peptide was analyzed using SignalP 4.0^48^. Disordered regions were investigated using IUPred^49^.

### Phylogenetic Analysis

To find homologous CPAP3 proteins in other crustaceans, homologous proteins were searched in the following five NGS libraries: (1) A transcriptome library from the Pacific white shrimp *Litopenaeus vannamei* composed of a wide array of developmental stages [published by Wei *et al*.^31^]; (2) a transcriptome library from the Chinese shrimp *F*. *chinensis* composed of several individuals at 15 days post larvae stage (Li, S., personal communication); (3) a transcriptome library from the ridge tail prawn *E*. *carinicauda* composed of several adult individuals with a body length about 5 cm (Li, S., personal communication); (4) our transcriptomic library from the giant freshwater prawn *Macrobrachium rosenbergii* composed of a wide array of tissues and developmental stages^32^; and the last NGS library (5) the only publicly available crustacean-genome of the common water flea *Daphnia pulex* available online via: http://wfleabase.org/ and described by Gilbert *et al*.^27^. Homologous proteins were also searched against the non-redundant protein database of NCBI using BLAST. Finally, representative CPAP3 proteins sequences (after removing the signal peptide sequence) from different insects and the found CPAP3 proteins from *Cherax quadricarinatus* and other crustaceans were used to build a maximum likelihood tree using MEGA6 software^50^, using the poison model, uniform rates amongst sites, complete deletion of gaps/missing data and a very strong branch swipe filter coupled to a bootstrap test set on 10 × 10^3^ repetitions.

### Spatial and temporal expression

Spatial-temporal expression patterns of the different *CqCPAP3* genes in *Cherax quadricarinatus* were examined using RT-PCR. Total RNA was isolated from the molar-forming epithelium, carapace cuticle-forming epithelium, gastrolith-forming epithelium, hepatopancreas and abdominal muscle from males in four different molt-stages: inter-molt, early pre-molt, late pre-molt and post-molt; EZ-RNA Total RNA Isolation Kit (Biological Industries, Beit Haemek, Israel) was used according to the manufacturer’s protocol. First-strand cDNA was synthesized by reverse transcription using the qScript cDNA Kit (Quanta BioSciences, Gaithersburg, MD) with 1 µg of total RNA. Specific primers were used for PCR amplification (Table S1); PCR was performed with REDTaq ReadyMix PCR Reaction Mix (Sigma); using the following specific conditions: 94 °C for 1 min, followed by 35 cycles 94 °C for 1 min, 60 °C for 2 min, 72 °C for 3 min, followed by 10 min at 72 °C. *Cq18S* amplification served as a positive control.

### Protein purification from exuvia and gastroliths

Proteins were extracted from three different cuticular structures; the “general” cuticle (surrounding the entire body and referred to in this article as the “cuticle”), the molar teeth and the gastroliths. The cuticle and the molar teeth were taken from animal exuviates. The gastroliths were extracted from late pre-molt animals. All samples were washed in de-ionized distilled water, frozen in liquid nitrogen, ground to powder and then incubated with different solvents as described below. Two consecutive extractions were performed, each with a different two step protocol. In addition, the second extraction differed from the first in that it had an extra step with harsher conditions. The following procedures were performed for all the solvents: The powder was dissolved at a concentration of 1 g per 20 mL of solvent with continuous stirring at 4 °C overnight. The insoluble residue was precipitated by centrifugation (1,500 g for 20 min at 4 °C) and the supernatant was separated off. The precipitate was then dissolved in the second solvent. The solvents used in the first extraction were: 0.02 M ammonium acetate, pH 5.0, that included 0.5 M EGTA, followed by 0.02 M ammonium acetate, pH 5.0, with 6 M urea. The first two steps of the second extraction were similar steps but they were followed by a stronger solvent extraction step, i.e., 0.02 M ammonium acetate, pH 5.0, that included 5 M guanidinium thiocyanate. All final supernatants were dialyzed twice, using a Cellu Sep dialysis bag, with a 6,000–8,000 Da cut-off (MFPI, Seguin, TX, USA)— first against 5 L of 200 mM ammonium acetate, pH 7.0, and then against 5 L of 20 mM ammonium acetate, pH 7.0, at 4 °C overnight. The samples were concentrated, using Vivaspin 20 (MWCO 7,000; Vivaproducts, Inc. Littleton MA, USA), yielding three fractions: an EGTA-soluble fraction, a urea-soluble fraction, and a guanidinium thiocyanate-soluble fraction. Protein concentration in each sample was determined by the Bradford method using Bio-Rad reagent (Bio-Rad, Berkeley, CA, USA). The protein profiles of the soluble fractions from the three cuticular structures were separated on an SDS-PAGE 4–12% Tris–glycine gel (Genscript, Piscataway, NJ, USA) using MES buffer, pH 5. The resulting bands were visualized by Coomassie brilliant blue staining, excised from the gel, and analyzed by tandem mass spectrometry, as described below.

### Mass Spectrometry

Extraction of the fragments from the gel, mass spectrometry, and data analysis were performed according to Shechter *et al*.^18^. The reduction, alkylation and trypsinization steps were carried out as previously described^51^. The tryptic digest was separated on a homemade column (15 cm long, 75 µm internal diameter fused silica) packed with Jupiter C-18, 300 Å, 5-µm beads (Phenomenex, Torrance, CA, USA) and connected to an Eksigent nano-LC system (Eksigent, Dublin, CA). The peptides were eluted with the following solutions: buffer A was composed of 2% acetonitrile, 0.1% formic acid, and buffer B was composed of 80% acetonitrile in 0.1% formic acid, in nano-pure water. A linear gradient of 20–65% of buffer B was created over 45 min. MS peptide analysis and tandem MS fragmentation were performed using an LTQ-Orbitrap (Thermo Fisher Scientific, San Jose, CA, USA). The mass spectrometer was operated in the data-dependent mode to switch between MS and collision induced dissociation tandem MS of the top six ions. The collision-induced dissociation fragmentation was performed at 35% collision energy and 30 ms activation time. Proteins were identified and validated against an internal database containing the predicted best translation of all the contigs from the molt-related transcriptomic library described above^10^; for this purpose the Sequest algorithm operated under Proteome Discoverer 1.2 software (Thermo Fisher Scientific) was used. The following search parameters were used: enzyme specificity trypsin, maximum two missed cleavage sites, cysteine carbamidomethylation, methionine oxidation, and a maximum of 10 ppm or 0.8 Dalton error tolerance for full scan and MS/MS analysis, respectively. Protein identification criteria were defined as: a minimal score of >50, a minimum of two peptides, and a false discovery rate (FDR) with a P-value < 0.01.

### dsRNA production and silencing efficiency test

CqCPAP3A, E, B1 and B2 were selected for silencing with RNAi. Two PCR products were generated using a T7 promoter anchor attached to one of the two primers used to amplify each product. The primers used for generating the template for *CqCPAP3A* sense-strand RNA synthesis were ds *CqCPAP3A*-F+T7 as the forward primer and ds *CqCPAP3A*-R as the reverse primer. The primers used for generating the template for anti-sense strand RNA synthesis were ds *CqCPAP3A-*F as the forward primer and ds *CqCPAP3A*R+T7 as the reverse primer. The process used for generating the template for *CqCPAP3E*, *B1* and *B2* sense-strand RNA and anti-sense RNA was performed as similar to *CqCPAP3A*, with specific primers as shown in Table S1. PCR amplicons were separated and visualized as previously described^52^. dsRNA was prepared, hybridized, quantified and maintained as described previously^52^. For evaluating gene silencing efficiency, *Cherax quadricarinatus* males (5–10 g) were divided into three groups; ds*CqCPAP3A* and ds*CqCPAP3E* injected (n = 5), ds*CqCPAP3B1* and ds*CqCPAP3B2* injected (n = 5) and as control an exogenous ds*RNA* (ds*GFP*) injected group (n = 5). The concentration of ds*RNA* was 5 µg/g body weight for all groups. First, all groups were endocrinologically induced to molt as described above. Animals reaching an MMI of 0.02 were injected daily with ds*RNA* until an MMI > 0.1 was reached. At this stage, all animals were dissected and mRNA was extracted from the cuticle- forming epithelium and the abdominal muscle as described above. The relative expression of the different *CqCPAP3* genes from the different treatment groups was evaluated using qPCR. First-strand cDNA was synthesized as described above. Relative quantification of transcript levels was determined using Roche Diagnostics FastStart Universal Probe Master Mix (Basel, Switzerland) and Roche Universal Probe Library probes. *Cherax quadricarinatus* 18S, which served as a normalizing gene, was also quantified by means of qPCR. Statistical analysis was performed using non-parametric tests as follows: For relative transcript levels between the different treatment groups, the Kruskal-Wallis rank sum test was performed, followed by multiple pair-wise comparisons using the Wilcoxon rank sum test. Differences were considered statistically significant at a *p*-value < 0.05.

### Phenotypic effects of the RNAi experiments

Two loss-of-function experiments using RNAi testing for phenotypic effects were conducted. A first experiment termed the ‘post-molt experiment’ was conducted on early pre-molt males (5–10 g), which were endocrinologically induced to molt as described above. At mid pre-molt (MMI ~0.06) these animals were divided into three groups: co-silencing of ds *CqCPAP3A* and ds *CqCPAP3E* injected (n = 7), co-silencing of ds*CqCPAP3B1* and ds*CqCPAP3B2* injected (n = 7) and, as control, exogenous ds*GFP* injected (n = 8). The concentration of ds*RNA* was 5 µg/g body weight for all groups. Injections were given daily until the animals reached ecdysis +7 days. The survival of the animals was monitored on a daily basis. Statistical differences in survival between the three groups were determined as described previously^21^. The second experimental procedure, namely, the ‘pre-molt experiment’, was conducted on inter-molt males (3–6 g) that were endocrinologically induced to molt as described above. In this experimental procedure, injections of dsRNA, 5 µg/g body weight, were started in parallel to molt induction. The animals were divided into two groups: co-silencing of ds*CqCPAP3A* and *E* injected (n = 9) and, as control, exogenous ds*GFP* injected (n = 8). The injections were continued until animals reached the onset of ecdysis, as defined by the morphological method of Drach as the end of the D_4_ stage^53^. The newly formed cuticle was then extracted and air dried. To test for differences in the structure of the newly formed cuticle, three values, namely, cuticle thickness, epicuticle thickness, and number of chitin lamellas in the exocuticle, were measured and then normalized against carapace length to compensate for differences in animal size. All the parameters were averaged based on four random points in the carapace cuticle and calculated using image J^54^. For the imaging, gold-coated samples of cross sections of the carapace cuticle were characterized by a JEOL JSM-7400f scanning electron microscope in the Ilse Katz Institute for Nanoscale Science & Technology, BGU.

### rCqCPAP3A expression and purification

The *rCqCPAP3A* gene was synthesized, digested with NdeI and XhoI and cloned into the respective sites of plasmid pET28a(+). In this construct, the *rCqCPAP3A* was fused in-frame to express a six-His tag at the N-terminus of the protein followed by a thrombin proteolysis site (performed by Biomatik, Cambridge, Ontario, Canada). *Escherichia coli* Rosetta strain cells harboring the *rCqCPAP3A* plasmid were grown in LB medium containing kanamycin (100 mg/mL) and chloramphenicol (30 mg/mL) at 37 °C. When the optical density of the culture reached 0.6 OD at 600 nm, isopropyl-d-thiogalactopyranoside (IPTG), 0.5 mM, was added to induce expression for an additional 16 h at 21 °C. The cells were harvested by centrifugation at 7438 g for 8 min at 4 °C. rCqCPAP3A-expressing cells were suspended in lysis buffer (phosphate buffered saline, pH 7.6, 20 mM imidazole, 1 mM Tris (2-carboxyethyl) phosphine hydrochloride, 0.02% Triton X-100) and incubated with DNase I (10 mg/mL) and protease-inhibitor cocktail [100 mM phenylmethylsulfonyl fluoride (PMSF), 1.2 mg/ml leupeptin and 1 mM pepstatin A] for 30 min at 4 °C. The cells were then disrupted by two cycles in a French press pressure cell at 172 MPa. Cell debris was removed by centrifugation at 19,000 g for 90 min at 4 °C, and the supernatant was filtered through 0.45 μm filter membrane to remove cell debris and other impurities. The protein was purified by immobilized metal-affinity chromatography (IMAC) on a Ni Sepharose High Performance column (GE Healthcare Biosciences, Pittsburgh, PA, USA) and was eluted with a 20–500 mM imidazole gradient in lysis buffer. The eluted rCqCPAP3A protein was concentrated using a Vivaspin-4 (3000 molecular-weight cutoff; Sartorius Stedim Biotech, Goettingen, Germany) and applied onto a size-exclusion column (Superdex 200 10/300 GL, GE Healthcare Biosciences), pre-equilibrated with column buffer (10 mM Tris–HCl pH 7.6, 200 mM NaCl, 1 mM Tris (2-carboxyethyl) phosphine hydrochloride, 5 mM N-acetylglucosamine) and then concentrated to 5–10 mg/mL.

### Circular dichroism (CD) spectrometry analysis

Circular dichroism measurements were conducted with a J750 Spectropolarimeter (Jasco Inc, Mary’s Court, Easton, MD) equipped with a Pelletier device. A sample of the rCqCPAP3A protein was prediluted to 6 μM in a buffer comprising 10 mM Tris, pH 8.0, and 200 mM NaCl and measured in a 0.1 cm optical path Suprasil quartz cuvette (Hellma GMBH & Co., Müllheim, Germany). Spectral profiles of the samples were obtained at a wavelength range of 190–260 nm at ambient temperature with bandwidth set to 1 nm, a scan speed set to 10 nm/min, and a time constant of 4 s. Deconvolution was performed using CDNN software, with a net set of 33 base spectra.

### 3D structure characterization using SAXS

Measurements were performed using a SAXSLAB GANESHA 300-XL system with Cu Kα radiation generated by a sealed microfocused tube (Genix 3D Cu-source with integrated monochromator) powered at 50 kV and 0.6 mA, and three pinholes collimation. The scattering patterns were recorded by a Pilatus 300 K detector. The scattering intensity I(q) was recorded in the interval 0.012 < q < 0.7 A^−1^, where q is defined as $q=\frac{4\pi }{{\rm{\lambda }}}\ast \,\sin \,{\rm{\theta }}$ in which 2ϴ is the scattering angle, and λ is the radiation wavelength (1.542 Å). A 1.75 mg/ml solution of rCqCPAP3A in 10 mM Tris, 200 mM NaCl, 5 mM N-acetyl glucosamine pH 7.6 buffer was sealed in a thin-walled capillary (glass) of about 1.5 mm diameter and 0.01 mm wall thickness; measurements were performed under vacuum at 4 °C. The 2D SAXS images were azimuthally averaged to produce one-dimensional profiles of intensity, *I* vs. *q*, using the two-dimensional data reduction program SAXSGUI. The scattering spectra of the capillary and solvent were also collected and subtracted from the corresponding solution data. No attempt was made to convert the data to an absolute scale.

### SAXS data analysis and envelope model

The GNOM program was used to obtain pair-distance distribution functions and the corresponding maximum dimension of the protein complexes (Dmax), as described by Svergun^55^. An *ab initio* envelope was generated by the program DAMMIN Svergun^29^, using atomic radii set to the dummy atom packing radius determined by DAMMIN without imposing a symmetry operation. The generated envelope model (DBM) was fitted on the core structure of the deposited solution NMR (1DQC) using Coot software^56^ and visualized by PyMOL^57^. The core residues of the 1DQC ensemble were defined with OLEDARDO^58^.

### Data availability statement

The datasets generated during and/or analysed during the current study are available from the corresponding author on reasonable request.

## Electronic supplementary material

Supplemental information
