# Supplementary material for: CPAP3 proteins in the mineralized cuticle of a decapod crustacean
Source: Sci Rep. 2018 Feb 5;8:2430. doi: 10.1038/s41598-018-20835-x (PMC5799365; doi:10.1038/s41598-018-20835-x)

CPAP3 proteins in the mineralized cuticle of a decapod crustacean

Shai Abehsera[1, 2], Shir Zaccai[1], Binyamin Mittelman[1, 2], Lilah Glazer[4], Simy Weil[1, 2], Isam Khalaila[2, 3], Geula Davidov[1, 2], Ronit Bitton[1,2], Raz Zarivach[1, 2], Shihao Li[5], Fuhua Li[5], Jianhai Xiang[5], Rivka Manor[1,2], Eliahu D. Aflalo[1, 2] & Amir Sagi[1,2]

[1]Department of Life Sciences, Ben-Gurion University of the Negev, Beer-Sheva, Israel. [2]The National Institute for Biotechnology in the Negev, Ben-Gurion University of the Negev, Beer-Sheva, Israel. [3]Department of Biotechnology Engineering, Ben-Gurion University of the Negev, Beer-Sheva, Israel. [4]Department of Psychiatry and Behavioral Science, Duke University Medical Center, Durham, USA. [5]Key Laboratory of Experimental Marine Biology, Institute of Oceanology, Chinese Academy of Sciences, Qingdao, China.

Corresponding author: Amir Sagi, P.O. Box 653 Beer Sheva 84105, Israel

sagia@bgu.ac.il, +972 86461364

Supplementary Tables

Table S1. Names and sequences of the different primers used in this research

**Primer**	**Sequence** 5'- 3'
T7P	TAATACGACTCACTATAGGG
ds*CqCPAP3A*-F	TGAGGGCTCTTCCCTACAGA
ds*CqCPAP3A*-R	AGATTAGTCCGCCAGCCTTT
ds*CqCPAP3A*-F+T7	T7P-TGAGGGCTCTTCCCTACAGA
ds*CqCPAP3A*-R+T7	T7P-AGATTAGTCCGCCAGCCTTT
ds*CqCPAP3E*-F	ACCCAGAACTTCACCTGTCC
ds*CqCPAP3E*-R	CCTCCTCCTCTGTGGTCTGT
ds*CqCPAP3E*-F+T7	T7P-ACCCAGAACTTCACCTGTCC
ds*CqCPAP3E*-R+T7	T7P-CCTCCTCCTCTGTGGTCTGT
ds*CqCPAP3B1*-F	TGCTGATCCAACAGACTGTCA
ds*CqCPAP3B1*-R	GTCGCTTTGGAACCGTGT
ds*CqCPAP3B1*-F+T7	T7P- TGCTGATCCAACAGACTGTCA
ds*CqCPAP3B1*-R+T7	T7P- GTCGCTTTGGAACCGTGT
ds*CqCPAP3B2*-F	TGAAGCTCTTAGTCTGCCTACTG
ds*CqCPAP3B2*-R	GCAGGAGAACCCAAGGTAGG
ds*CqCPAP3B2*-F+T7	T7P- TGAAGCTCTTAGTCTGCCTACTG
ds*CqCPAP3B2*-R+T7	T7P- GCAGGAGAACCCAAGGTAGG

Table S2. Accession numbers of newly found CPAP3 proteins in different crustaceans.

CPAP3 name	Accession number
MrCPAP3B	MF407550
MrCPAP3E1	MF407551
MrCPAP3E2	MF407552
LvCPAP3A1	MF415537
LvCPAP3A2	MF415538
LvCPAP3B1	MF415536
LvCPAP3B2	MF415535
LvCPAP3E	MF415539
LvCPAP3I1	MF415540
LvCPAP3I2	MF415541
LvCPAP3J	MF415542
LvCPAP3A1	MF415537
FcCPAP3A	MF415532
FcCPAP3B1	MF415531
FcCPAP3B2	MF415530
FcCPAP3I	MF415533
FcCPAP3J	MF415534
EcCPAP3A1	MF415523
EcCPAP3A2	MF415524
EcCPAP3C	MF415525
EcCPAP3D	MF415528
EcCPAP3E1	MF415526

EcCPAP3E2	MF415527
EcCPAP3J	MF415529

Supplementary Figures

Abehsera et al Figure S1

CqCPAP3B1

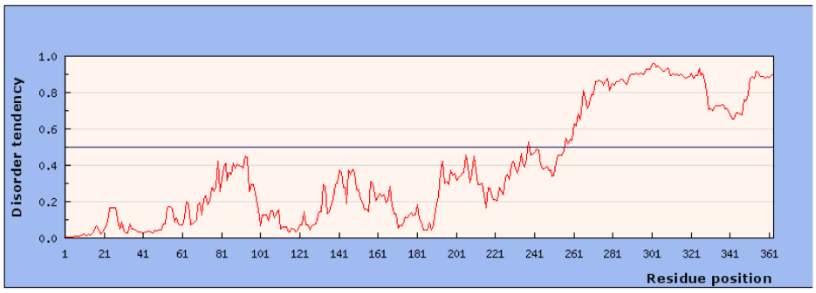

CqCPAP3B2

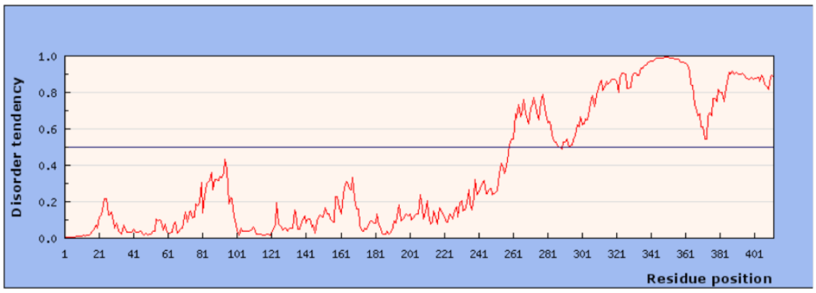

CqCPAP3J1

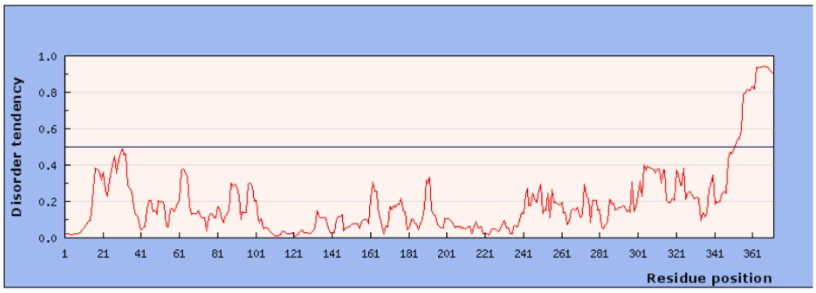

CqCPAP3J2

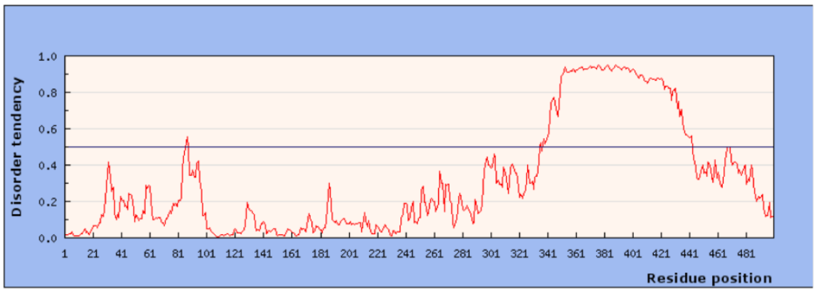

Figure S1. Prediction of intrinsically disordered regions by the server IUPRED in different CPAP3 from *C. quadricarinatus*.

Abehsera et al Figure S2

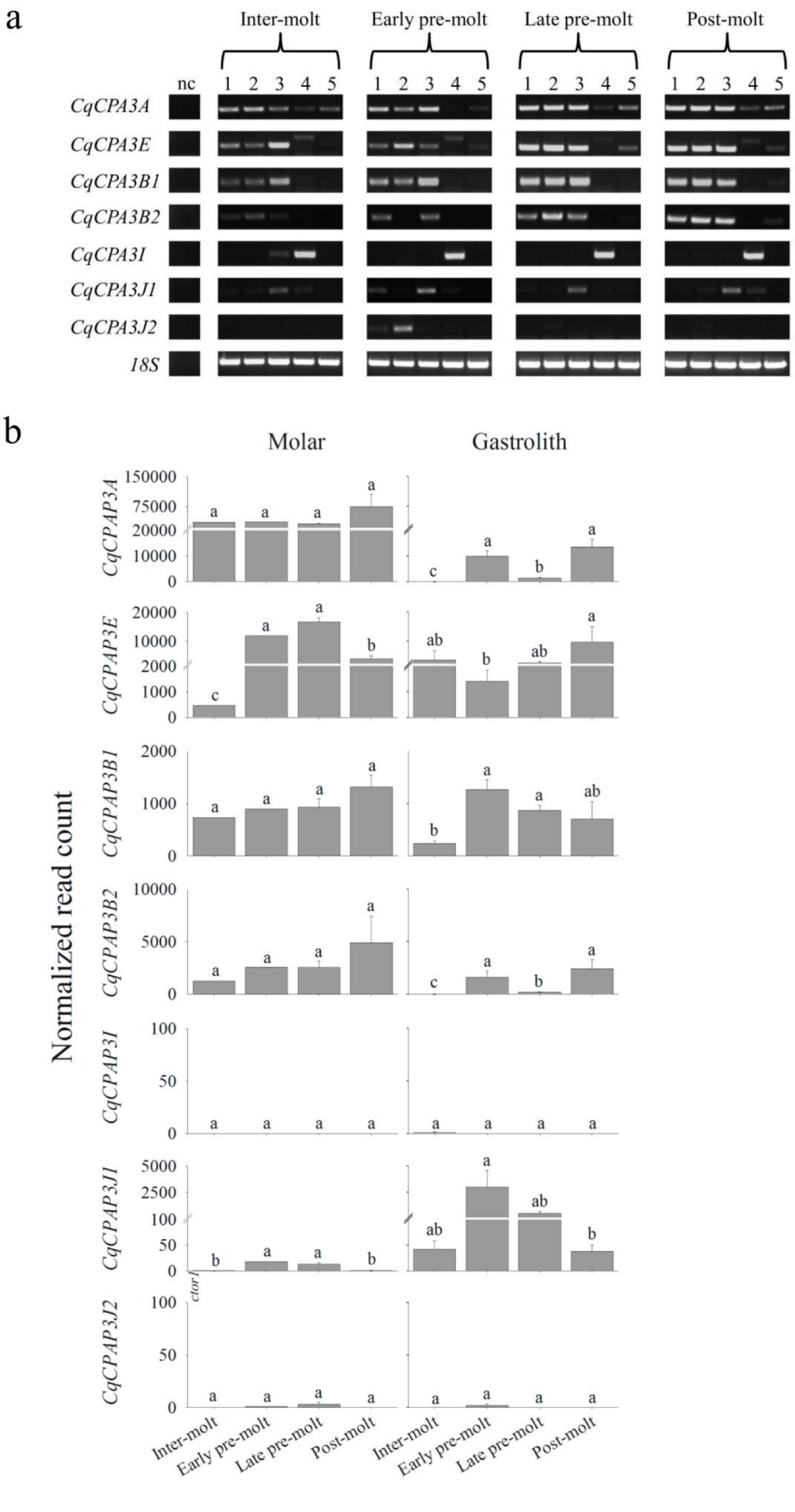

Figure S2. CqCPAP3A, E, B1 and B2 are the most active CPAP3 proteins in the cuticular-forming epithelia of *Cherax quadricarinatus*. a) *In vitro* spatial-temporal expression of the seven *CqCPAP3* transcripts found in *Cherax quadricarinatus*. For each transcript, spatial-temporal expression is shown in five tissues sampled in animals from four molt stages. NC- negative control, 1- molar-forming epithelium, 2- carapace cuticle-forming epithelium, 3- gastrolith-forming epithelium, 4- hepatopancreas, and 5- abdominal muscle. b) *In silico* spatial-temporal expression based on data from our molt-related transcriptomic library showing normalized read count of the seven *CqCPAP3* transcripts found in the molt-related transcriptomic library of *Cherax quadricarinatus* from the molar-forming epithelium (left) and gastrolith-forming epithelium (right). Letters represent statistical groups that are significantly different (p-value <0.05); error bars represent standard error.

Abehsera et al Figure S3

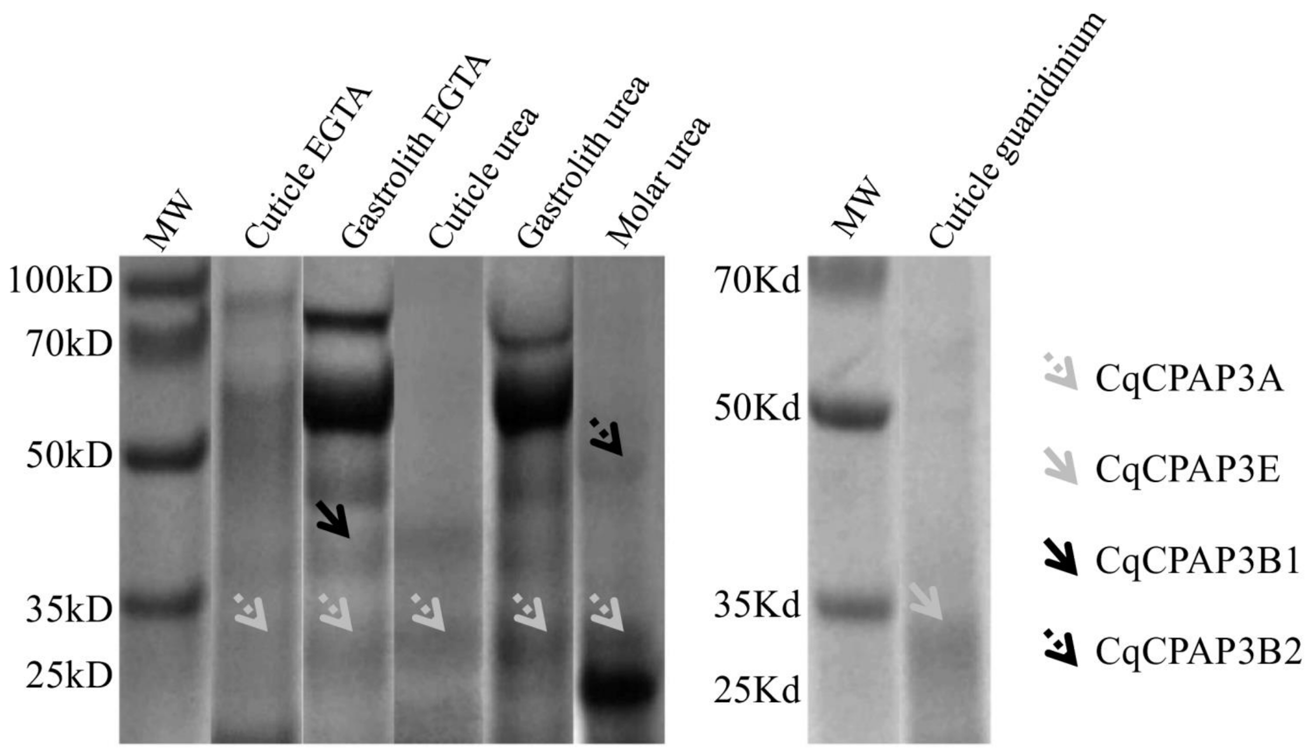

Figure S3. CqCPAP3 proteins are found in the mature cuticle of *Cherax quadricarinatus*. Coomassie staining of the SDS/PAGE-generated protein profile of the extraction of proteins from different cuticular structures and soluble-fractions in which CqCPAP3 proteins were found. Arrows point at the location of different CqCPAP3 proteins.

Abehsera et al Figure S4

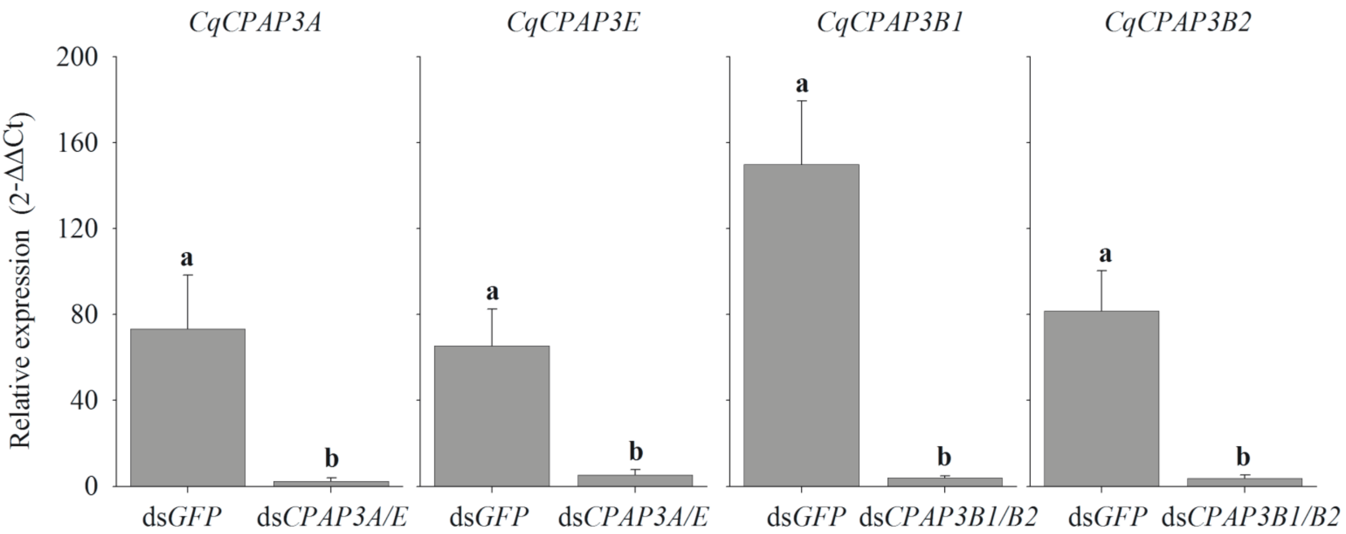

Figure S4. Relative expression levels following dsRNA-injections. Relative expression levels of *CqCPAP3A, E, B1* and *B2* (right to left) transcript in different dsRNA-injected groups, dsGFP-injected (n = 5) as control and either ds*CqCPAP3A/E* -2 (n=5) or ds*CqCPAP2B1/B2* (n = 5). Different letters represent statistical groups that are significantly different (p-value <0.05); error bars represent standard error.

CPAP3 protein sequences used in the phylogeny study

>CqCPAP3-A

QFQCPPDDGFYVDPRQCDKYYDCYRGTQTEKLCPDGLIFDTTQGPRVEQCNYPFIVECPEGSSLQTPQPS
GIECPRQNGYFEHENAKNCESYYECTNGLAVLRKCATGLVFDEFTGVCQWSHTGVRSGCGFVPEVLADG
FSCPNNTQLHTNGQELDHARYVNPNDCRFFYVCEDGIHPREVGCPKGTVFNDVNLLCDAPENVPGCEN
YYPAEDVRSAKAGGLI

>CqCPAP3-E

WGQSCPPEDVHYFSDSQQCDKYVECRDGVATEQLCPDGLLFNDKIKNGRYPCDYPSEVDCGSRGRTQP
PQPTEFCQHQWGYFGSGNRAECGYFYNCVNGVAHLVNCPAGLAFSSSTYRCEWADESSDCDAEAYLGF
SCPNSVDTNQLLLSGYPQFRSPRDCRQFFICVNTSPRLQFCQLGLVFNEETYTCDEPQNVRGCENYYTPEE
LALYREQRERQRIAEEKRQAELDTLRQQLAARRKQQLQ

>CqCPAP3-B1

LCDGQELASECPAPNGYFADARQCDRYYECVDNVMSVRLCPDGMAFNDFNPAVEKCDLLSQVDCTGR
PDLQPARPTEHCDRQYGNFAPKETGNCQSAYRCVDGVGTLISCPEGLAFSLETGICDWPDQSGRKDCDK
QKTQNFTCPKVNFDIAVSHPRYADPHDCQYFYVCINGDIPRRNGCAFGQVFNTNTTTCDSPKEVPECAD
YYTEYFDEYFRTLEKDPGKMSADIVAAAVAAGYDVPKFRDRVRINPGAATPLRRGTTSPPVVPRTEEPRLR
PLAGKPGESRDGKPGDRPQRRRPGGIRRKRPTLTTTTTTTTTPAPDDYEYYYDYPEGSEATAAAPGPSRT
TLPI

>CqCPAP3-B2

VEAQGVVPECPAPDGFFADAEQCDRYYECVDNVLTEKMCADGLAFVDLNPRIEKCDYISSVDCTGRPKL
QPAQPTDVCPRQNGYFQHPDETVCNKFYFCNAGTGNLVTCPEGLVYGLDSHNCVWPDVAARQGCSSD
VIAEFDCPKVTDDIAVSHPRYADPTDCQYFYVCISGVNPRRNGCAFGQVFNSKLAACDAPQEVPECADYY
TAYFEDYFNTLTATDGRVSVDILAAALASGYDVPSIQERFHVDPKLLQTQPRPSRARGVTAAPAAAAPAA
VAPAAATVRKAGKLPARRQEELVSQDGDVSPPSLTAGAPRNTVPKRRFRPQPPTTTTTPPPPPPPPQGG
PEDYTDYTDYTDGAVYDYGAEVPLPPPTTTTAAPVQPTSIKRRIPLRRPRPAN

>CqCPAP3-I

IGPSLQLCVPDCTGRAEGDMIKDPSNCLQYYFCSDPDGNGVLEHSLYPSTCPEGYFFNAAESVRECEKIVP
DTNYCKDLCSPRAIQCQEPGTMLPSPTDCQLYKICLDNGFLDVSCPPEYPNYNFETSDCTNDDTVCYNLC
DPCEVYCVAEGKIPDPKNCNGYLYCHPPEVAYFLCPEGEAFNSDLLLCEPFAGNCTNTC

>CqCPAP3-J1

LVGVVTPQEEEQRPLSMRCVSEGRFPHPSDCGGFVDCLPDGEGLRAREGSCRGRAYHPTLRKCVTLNKV
DGCKPRAARALANDPKLDYVCENSTSDFMCADCKTLVLCVNGTAYPETCGSGDLCNIDNDHFGGGVCY
PNQPSDCSCDAPNQFKEDSYNEARFLFCEATGSDPQIFQCPDDHVFDSTMNQCIHYNGLPECTSIGVFA
NSRNCSQFYNCIPTIDGWVQKPFSCNNETHKHLMYNEAAGSCEDPCTWGNGNFKCEEEGRFADPLNC
QRYYECVKDTSEASDFRQALHECPEGYEWDQSARNSYGHCVLEGNSLNPCVPAVANKCSVDRSQCPSTT
EEPDDTTDQPTTRQHE

>CqCPAP3-J2

CFWLQEKNNLECERAGRFPDPSYPGGYIDCVMGSSGLTARPGSCKGAEYNSELRKCLSVSKGHHVYKEES
SKTGARQQPDDLCSDIVNGFVCSDCKTLVLCLEGEAYTEPCDAGDFCDVREEDFGGGVCYPNWDTVCSC
SDEQCFSRDVYDDQKFVYCWPDVDKADVYRCPDGMRFSKEQQKCVSTIGNITCAQNGVFANPDDCTEY
YDCVYTDIGWKTRIHKCSDGLMLNENTGNCEDPCTWPTGDFSCTEEGRYPDPLDCGRYYVCIAEGEGFR
QLQRQCPELYYWDPSEKTGQGICVKATISTCSPISASRCQVPEEWCVSYPSDQDTEVDQDAEADRTTDA
DQTTDEDQTTDEDQTTDADQTTDADQTTDEDQTTNEDQTTNADHSTNADHSTNADHSTDTDQSADA
DHSTKVDTHALPSGCELPLLGAGRDCLFITGEATQATDFARECEIAGGEAVSLTDSHIQAIQDYIDPVLLKN
VGVWVTGPFSITRKFLWPDGTRVNMRQVMGTTWDHDSFCLYITSNIKFPFSLGYCNHFKRVLCKVRVK

>MrCPAP3-B

CDFLFQVSCKGRPDLQRPKPTDNCPRQNGYFPHEDQTNCREFFYCTNGISSPLTCPESLVFSTKTGTCVW
PEEAGRTNCASEKVFNFTCPKVGMDTAVTHPRYADPQDCQYFYVCINGETPRRNGCSFGQVFDTKSVTC
AEPKDVPDCADYYIEYFENYFETLEAGARPSGDILTAARASGYEIPEFRERVRIRPGDAVPTRRGTTTASPR
GERPEGRPLRRRRPGGNGGLRRRKRPTTTTTTTTTTPPPEYYDDYYYYDETYDNVDNKDDSSY

>MrCPAP3-E1

AQSCKDDEILYYPDSQECDKYTQCKFGVVTEEYCPPGLLFNDKVTNGRYPCDYPIDVDCGSRSRTQPAVSS
ENCPNAWGYYGTGDRANCGYFFNCVDGQAFPFTCPEGLAFSSATYRCEWPDESPDCDSSAFLGFTCPE
QQDPTQLLLYGHARLRSPRDCRQFFICVGPSPRLNHCELGQVFNEAANSCDEPENVAGCENYYPREELAA
IRDRKEKQRIAAAKRQEDFEQLRQQLVRRN

>MrCPAP3-D

AQRGREAAQPSYPTYREITSCPELEGLQLYPHPASCHQFYKCANGTLTLETCENGLLFDGKGAVHNYCNY
HWGVECEGREFELVPIARGICEFAFGLYSSGQCEKYFVKCEYGEPIDVPCTAGLAYDERIHGCNWPDLLEY
CNPEDVVGFKCPAYAEPGSLSAQFEPFPRYPSGDCGRYIVCVDGYPRLTGCGDYTVFDEETLTCQDPEYV
PKCANYFKK

>FcCPAP3-A

AQFQCPAEDGFYPDNKQCDKYYDCYRGTMTEKVCPDGLVFDIAQAPKVEQCNYPFLVECGEGSTLQIAQ
PSGIECPRQNGYFEHEDPSNCAQYYECTNGEAVLRTCATGLVFDEFSGTCQWASAGFRTGCGERVEVLP
DGFSCPNETQVHTNGQALDHTRHVKPSDCRFFYICNEGKYPREVGCPQGTVFNDNTLNCDDPRNVPGC
ENYYPDDDVSAVARAGINI

>FcCPAP3-B1

CLSQEASDACPAANGFFADAVQCDRYYECRENEIEDKLCPDGLVFSDKNSKLERCDFPFNVDCGDRPELQ
DPQPSTNCPRKNGYFPHRDPSVCDQFFFCSDGQFNLITCSPGLVFDAKTGTCAWPGEANRVGCSGKDV
NKFTCPEPLPNDGGVVNPNPLYADPEDCQHFYVCINNVEPRRNGCPLGYVFN

>FcCPAP3-B2

GSVRAQSVASECPTPNGFFADSEQCDKYYHCLDDVLTEKVCPDGMAFNDLNPKVEKCDFLFQVNCEGR
PRLQNAKPSENCPRQNGYYPHPEPTNCHQFYYCTGGQSSQLTCPEGLSFSVNSGTCVWPDQAGRSNCV
SEKLLNFTCPALPPSSTEVHPRFADPDDCQYFYVCINGKEPRRNGCAFGQVFNNMTKTCDAPKDVPECK
DYYTEYFDNYFSTLNPEQGGRVSTDVIAAAIASGYPVPRHRNRVHVQPSQAGQGDAPSRPAAEEEPEAP
RRPVAPVRRRRPGGGVRRRGRPRTTTTTTTTTPEPDYYYYYDDYYNDENYPEEAAPDTEPAKPPSATRNT
LPILSRN

>FcCPAP3-I

GLGFCHGKLCIPDCSEMREGDKVPDPTNCFRYYYCSDPEGTGELIHSSEPLDCPEGYYFNAELHILECEPIV
PENMHCTALCNPCEVKCTAPGTLIPNVNDCGMYKICLDDGSSIDDYCPNNLPYFDYEAGLCSQDPGNCY
SLCDACDLCTHEGKVPDPHDCTMYYYCDPPLLSHFECPNDETFNPDTLVCEKNPTGNCTNLC

>FcCPAP3-J

PDRFLCLNCKTLVVCVRGQAFVRHCIEDHYCTDKSEFGGGVCYPNEPVECTCETPYKFRVDHYDPQRFFA
CSDVGSKPESYKCPDGMVFDENITQCRNEAGLPPCTKPGTFVNPENCSEYYSCIALRHGWLQKFFMCNG
NTFFNERKHICEDPCIYKFMCDSEGRFPDPVNKQRYFECYLESGQLKEMRYQCPQGYTWYNVSTGVGKC
VEDYGISRDDHNFDHCSLPQDWCPSNGIGPTVRGIPRTFRPEYEGDNIQTASASQDLNVIWVGSKSKN

>EcCPAP3-A1

LVTGLPQEIQFVSNTNSRGKDFICQEDYGVYEDEYQCDKYWVCEDGIAKAQLCDDGLVFDIFKANAGHV
DPCESPYVVDCGQRLELQPATFTSEVCPRKNGIFADPDSTNCIRYYTCIKGVATATDCTTGLHFDETTGNC
AWPSTANRSNCSELQNNCIGDFCCPGTPVVTADGVTLPHPTYANVEDCQKFWVCLSGVTPQESSCALG
QVYNERTMMCDYPEYVDECTGWYRDHPLFADDYVYEDTVPAKNQGTQNKAG

>EcCPAP3-A2

QFNCPGDDGFYPDPRQCDKYYDCYRGSMTEKLCPDGLVFDYTLSPGVEQCNYPFIVECPEGSALQPAQP
SGIECPRQNGYFEHEDPSNCEQYYECTGGVPVTRSCATGLVFDEFTGTCQWAHTGIRTGCGQRVEVLPD
GFSCPNNTQVHTNGQELDHARYVKPDDCRFFYICYEGKYPREVGCPQGTVFNDANLICDAPENVPGCEN
YYPNEPLTGLRAAGLGL

>EcCPAP3-C

AIGAQESSVSPGGAGNWQCPHEFGFYPHDTSCDKYYSCEAGVATLKTCGNGLAFDNSDSEYLRENCDYS
HNVDCSTRPQLEPAISTPHCPNLYGIFPDPDDCAVFWSCWDGEASRYACAPGLAYDRKSRVCNWMDNI
AECKTQRDAMEQTIQCPAPGQLAATGSFSRHAHPDDCRQYFVCLDGVPREYGCPIGTVFQIGQEDGFG
QCADPENVPGCSDYYGDLDLNALRRSQLVLGNIGAQDDQSGSAAAPAAPRPRRPSAPAQPQTVDVAEE
EVFQE

>EcCPAP3-D2

AGAGLAQQQQQPDPCDRKSRVVGDITYCDRYWECVAGQPQLYDCPNGLVFVGRNRGIAEGCDYPWR
GAYCDTKQLANPPISTEHCDWLYGIFGHETSCTRYWTCWNGTATEQFCIGGLLYNEETHACDWPQNVG
GCQKHPLCKDDPNANVPLGKSCERYWACQGGYPRLQRCPATLVFDKQSRRCVNPPTVDCDVPSTTPPP
EEERSGEPVQPRPQRRRPTLQAAPVRETFIPENAIPVAPQGLRPIPQEPAFPVPDARPLPSGGNFRPAPIP
GAIPLQ

>EcCPAP3-D1

AQRGRQTAEPAYKAYPEITSCPEVYGLQLYPHPASCHQFYKCANGTLTLETCENGLLFDGKGAVHNYCNY
HWGVDCEGREFELVPIARGICEYSFGIYSTGQCEKYYTKCEYGEPIDLPCTPGLAYDDRIHGCNWPDLLEYC
NPEEVVGFKCPAYSEPGSLSAQFEPFPRYPSGDCGRYIVCVNGYPRLTGCGDYTVFDEETLTCQDPEYVPK
CANYFK

>EcCPAP3-E

AQSCKDDEILYFPDSQECDKYTQCKYGVVTEEYCPPGLLFNDKITNGRYPCDYPVDVDCGSRTRTQPAVA
SENCPNAWGYFGTGDSSNCGYFFNCVDGQAFTFTCPEGLAFSSATYRCEWPDESPDCDSSAFLGFTCPK
EQDPAQFLITGHARLRSPRDCRQFFICVGPSPRLNFCDLGLVFNEATSSCEEPETVAGCENYYPREELAAIR
DRKEKQQIAAAKRQEEFAQLRQQLARRN

>EcCPAP3-J

TARSLTVDRRFSSICDSRRNGFFCADCKTVVNCIEGVAFVENCGTSDTCSIKDAFGGAICYPTPEPSCSCSR
ANAFFIDPYDTQMFFFCSATEAEPDMYHCPGEMIFDESTQQCKNANGFSPCMEAGVFAYHDDCTQYYT
CIASQNGWIQKPFSCSDGLMYNEIKGQCEDPCSWDTGKFTCQREGRFSDPLSCDKYYECVLLSDGSFRQ
DQRQCPHGYEWVQSELGVGRCGKGPSPNCQPLTLTKCIIPDTCSEAPAGSVAPAGSAAPAGSAAPAGSV
APAGSVAPAELITNFRTSGGNSNPPQYAFVHYPVGSDSVWNPPNRGDRRARVSN

>LvCPAP3-A1

DFLCPKSDGIFPDPNQCDKFWECVDGEAESQLCPDGLVYDPTNRNVNKCDHRFQVDCSDRPELQPAQT
TEACPRRNGIFAHPESSVCHLFYTCVDGAAVQTECAAGLVFDENTGTCTWPESAGRQGCFKKAGRLPDG
FECPTEEVKDVRGLTEIHPKYPHEDCAKFYVCLNRIEPRLLTCDRFKVFNVEKSFCDDPENVPDCEDYYGDV
PVFQKPTTKVRE

>LvCPAP3-A2

AQFQCPAEDGFYPDNKQCDKYYDCYRGTMTEKLCPDGLVFDVAQAPKVEQCNYPFLVECPEGSTLQIAQ
PAGIECPRQNGYFEHEDPSNCNQYYECTNGGAVLRTCSTGLVFDEFTGTCQWEHAGYRTGCGKRVEVLP
DGFSCPNETQVHTNGQALDHTRHLKPSDCRFFYICNEGKYPREVGCPQGTVFNDATLNCDDPRNVPGC
EGYYPEDELTPVARAGIKN

>LvCPAP3-B1

CLSQEASDACPAANGFFADAVQCDRYYECRENEIEDKLCPDGLVFSDKNSKLERCDFPFNVDCGDRPELQ
DPQPSTNCPRKNGYFPHRDPSVCDQFFFCSDGQFNLITCSPGLVFDAKTGTCAWPGEANRVGCSGKDV
NKFTCPEPLPNDGGVVNPNPLYADPEDCQHFYVCINNVEPRRNGCPLGYVFNDDTKQCDDPANVPECK
DFYGEVEEK

>LvCPAP3-B2

SIGIVSSLLLACASAQQLVSDCPSPNGYFADSVQCDKYYDCVDDVLTEKLCPDGMAFNDLNPRIEKCDFTF
QVDCTGRPELQPAQPTEVCPRQNGYFPHPDPKTCNKFYYCAAGAGSLITCPDGLVFSLKTGNCVWPDAA
GRSGCASTNVLNFTCPTSGFDIQRDAHPRFPDPEDCQYFYVCINGKDPRRNGCAFGQVFNSVTKACDSP
KEVPECADYYSQYFDEYFQTLSSGGRVSADIIAAAIAAGYDVPDLKDRVRVATGGSGSGATRRRGQTPAP
PLDAADDTAFRAPAPAARRPPASRPAARDEQPQRASRPSRPRLSVRPKPTPPPTTTPPPPPPPAPVDDYA
YDYAYEDYPADALYDEPAPAPATAAPPPPPAPAATPNRGRVVNRRPLVRPRN

>LvCPAP3-E

AAAQNCPEEGVAYFADRDFCDRYSQCRDGVYTEEQCPDGLLFNDLVTNGRYPCDYPSEVDCGSRSKRQ
PAQPTEFCAHQWGYFGSGDRAQCGYFYNCVDGQAHQFACPDGLAFSSATYRCEWPDESPDCDSAAFL
GFTCPAEVDLRQVQLFGHSRLRSPRDCRQFFICVGASPRLNYCDLGLVFNEAISSCDEPENVQGCQNYYP
PEELAAIRDRKEKARLAQLKREEEFQQLRQQLAQRRQN

>LvCPAP3-I1

VGLGFCHGKLCFPDCAEMREGDKVPDPTNCFRYYYCSDPEGTGDLIHSSEPLDCPEGYYFNAELHILECEPI
VPENMHCTTLCNPCEVKCTTPGTLIPNVNDCGMYKICLDNGNSLDDVCPSNLPYFDYETGLCSQDSSACY
NLCDPCDLYCTHEGKVPDPVDCTMYYYCDPPLLSHFECPNDETFNPDTLVCEQSLTGNCTNLC

>LvCPAP3-I2

VSPSVQVCAPDCTGVDPGTKVRDPTDCTRYYVCVDATGSGVLVPSIDPVECPDGQYFNDQHTSPRCDPI
NSAPNGFCSQLCNPCEPHCTHAGEVTPDPLDCSTYYVCLQDDHFMSVGCPAETPYFDFMAGECQGDST
LCFHNCDVCEPHCTQQNERVPDPTDCHRFYLCTPPTMSSFLCPHNEIFNRETGVCEEGATCIEECGGGVT
DGI

>LvCPAP3-J

VVVKDVTCANGGESSSHSLTCTVEGRNSHPTDCTKYVECIRVGGKLTPRVGVCENSAFNPATRNCVSFRD
TACQQPRRQARALFEDNAFSYMCSEGKDGFFCADCKTLINCVDRHAYKLTCVNGDMCAHKESFGGGIC
YPKEPAECTCQEPNSFKEDLYDNRKFFYCNDTSSDPVFYECDEGSAFDAEKSQCKNKNGLPECHRSGVFA
DINNCTQYYACISTRNGWVQKAFSCNQSNLMYNDLTGACEDPCTWQVGSFACTAEGRYPDPKDCQRYI
ECVADEGGLKQVRRSCPEDYSWDPTARGGVGHCVKASTNTHCTPATQNKCIIPDGTCASAAVATAATP
APEPTSPSTLVVSAELNVATTTTEAAPTTPPKAPVARRRRLNRRLNN

>AmCPAP3-C

GATAQESFKCPDDFGFYPHHISCDKYWKCDNNVAELKTCGNGLAFDASDSKFLTENCDYLHNVDCGDRT
QLEPAISTPHCPRLYGIFPDEKKCDVFWNCWNGEASKYQCSPGLAYDREARVCMWADQVPECKNEEVA
GGFTCPAAGEVSGASGSFSRHAHPDDCRKYYICLEGIAREYGCPIGTVFKIGDADGSGACEDPEDVPGCE
DYYGDLDLKSIRKSELLAGIQNSGETRKPPQGKPRPPSAPARPNASLQE

>AmCPAP3-D

LPFLLSVEATTLLGAPPCPDPHGVFAYAHPENCNAFFLCTNGTLTLEYCENGLLFDGHGAVHDHCNYHW
AVHCGDRKADLTPISSPGCKYQFGLYPASDACSTTYIRCAHGHPNEDHCDAGLVYDAKSHNCVWPDQLL
PYCNPEEIVGFKCPHKVPSHSAAAKFWPYPRFPVPGDCGRLITCVDGNPRLLTCGDGKLFDSVSLSCLDPD
ELPHCANNNL

>AmCPAP3-B

VTGLTRKQEALEQNRRKVSSPVLQRKQQQQQQSAEEEYQEDEEEVSDRCPEPNGYFPDAGQCDKYYDC
RDGKYIEKLCPDGLVFNDFSPQHEKCDLPFGIDCSKRPKLQKPQPSPHCPRMHGYFAHEDTRICNTFYYCV
EGKFNMITCPEGLVFSEKTGICNWPDEAQKKGCGSRELFNFTCPRVDEAIAATHPRYPDTEDCQYFYVCV
NGEIPRRSGCKLGQAFDERTGKCDWARKIPECKDWYKGQLTDEELDALENPPPKPKPSSGGQHRRKGAK
PT

>TcCPAP3-B

GLAAAARKHQKQESRDEEYEATDQCPEKYGFFADAEQCDKYYECNDGQITEKLCPDGMVFNDYSSEYEK
CDLPFNIDCTSRPKLQEPQPSQHCPRKHGYFAHEEPHICDKFYYCVDGKYNMITCPNGLVYNDKAGICSW
PDEAKKKGCSSEEVFQFECPKVNETVAATHPRYADPDDCQYFYVCINGDTPRRSGCKLGQVFDDVGKKC
DWVRNVPECADWYKGRLTDEQLKELENPPTPKPRPTKVSRRKPRPPRPTQVEEEEEK

>TcCPAP3-D1

AGVVLQDAPSCPEQHGVQAYAHPESCNLFFLCTNGTLTVEQCENGLLFDGKGAVHHHCNYHWAVDCG
HRKADLTPISTPGCEYQFGIYEESHGCSTHYIKCAHGEPIPQECEPGLVYDERIHGCNWPDLKLEVCNPEA
VVGFKCPTKVPSNSPAAKFWPYPRFAVPGDCHRLITCVNGFPRLISCGEGKAFDQHSLTCEEPELVPHCA
NHIRK

>ApCPAP3-C

YQVHGQREEFKCPDDYGFYPHSLSCDKYWKCDNNVAELKTCGNGLAFDDTDPKYLKENCDYLHNVDCG
ARSQLEPAIGGPHCPRLYGIFPDDVKCDTFWNCWGGEASRYQCSPGLAYDRESRVCMWADQVPECKID
EVANGFNCPAAGELLASVGSFSRHAHPDDCRKYYICMEGTAREYGCPIGTVFKIGDSDGSGSCESPEDVP
GCEDYYGDLDLKAIRKSELLSGLDHKSKQQSSSAESKAAPAAPKPQPQRQQQQSNRPQPQQQQSSRPQ
PQQLPVSSQSSKDYEDA

>ApCPAP3-B

ALLSGQSTGGRKRPPTTTAAAPRYRPTAVEDEEAVDQQESECPEPNGFFADASQCDKYYACSDNKITEKL
CPDGMVFNDYSSQQEKCDLPLNIDCSQRPALQTPQPAEHCPRQNGYFAHENQNICDKFYYCVDGKFNA
ITCPGGLVYNEKSGICTWPDEAKKKGCSSQDVFNFRCPNVTSEIALQHPRYANPEDCQFFYVCVNGDTPR
RNGCKMGQVFNEASGKCDWPRNVPECADWYRGVLTDEELYNLENPKPKETTKEKEAAAARRKPSRPPT
TKRTVEEDDE

>TcCPAP3-E

TISIIVTIVTLIAIQGLAQRNLGPSSCPEKNGRYPTSTCDGYIECRDGLAEEKLCPDGLLFNPASGPQAFPCQY
PLDVDCTGREQTQPAQATDECPHQFGYFRMGDATSCGQFKNCVDGRGFIFDCPEGLAFNGDTYRCDW
PDQVATCDAEAFLGFTCPNDGRSFGLGEAEFRFFRSPNDCQRYFVCVNGRPRLYNCGEGRAFNDLIGAC
DGVENVTGCVGGAQGGANFRNYRI

>NvCPAP3-A1

VIAASHAAYSCPKEDGQYEDPKQCDKFYECIDGLPIEKYCPDGLVFDPLNRKINKCDHVFNVDCGDRLELQ
PPQPTKKCPRKNGFFAHPDPAVCNIFYNCIDGEAIEITCTTGLHFDEYSGTCVWPDSAGRKGCGVVGKTL
SDGFECPKDAGVDSRGLAVDHPKFAHPEDCQKFYVCLNGVTPREQGCSDGTVYNEVQQRCDAPENVP
GCEDWYKDDDKKP

>NvCPAP3-D1

AALALVATADAAALFGGDVPTCPEPWGVQAYPHPEDCGSFFLCTNGTLSLEHCENGLLFDGKGAVHNH
CNYNWAVHCGHRKADLTPLSSPGCEYQFGIYPDSDSCSTTYIKCAYGEPHQAHCDAGLAYDDKSHTCVW
PDQLIPYCNPEAVVGFKCPAKPPTGAAARFWPFPRFPVPGDCGRLITCVEGHPRLITCGEDKLFDSETLSCL
DKDELPHCA

>NvCPAP3-E

ICVLVCTCYAQFQRQTPAPQRRVNPFFQERVNQPPFNQFSQSQQNNFHQQQHPRSGGASCPEPNGRF
PVPTQCDAYIECIDGVGEEKLCPEGLLFNPEARFNYPCGYPIDVQCLGRSALQPAQPTEDCPHQFGYFKM
GDRTNCGKFMNCVDGRSYVFDCPEGLAYNPETYRCDWPDQVPDCDAEAFLGFTCPTQDPNSFLVSETR
FYKSPNDCQHYYICVDNRPRLQNCGAGHAFNELINACDAAENVTGCEPPQTFNEVQQVRLDQPQHQN
KLRTFQQKSQPQNAFIQQNSINQQPNYNQQQNYNQQQGFGAFNQPQSRRNQQARGQVHYQRQQY
NGF

>NvCPAP3-B

ATALATRKEEAAENNRRKNSQPFYRKQPARVAPTPAAPEDDAAVEEETAAPSVTSPLLTRKQEAAEINRR
KNNQPLLNRHPARVTSEDEYDEEEAGEFSDQCPEPNGYFPDAEQCDKYYDCRDGKLTEKLCPDGLVFND
FSPQHEKCDLPFGIDCSKRPKLQTPIPTAHCPRMHGYFAHEDPTNCNTFYYCVEGKFNMIKCPDGLVFSE
KTGICTWPDEAHKTGCGSRELFNFTCPKVDESVAATHPRYPDSEDCQFFYVCINGTPRRSGCKLGQAFDE
STGKCDWARKVPECKEWYKGVLTDAELDALENPPKAKTSGSSRRKGPKRGAEPAE

>Daphnia magna CPAP3-E

ASRCDKFLECRDGVAEEVTCPDGLLVNEKAAAFRYPCDYPNEAEEVTCPDGLLVNEKAAAFRYPCDYPNE
VDCGKRTEPPASPSYGNCARKWGMFKIGDSCGDFVNCVDGVEHQFKCPEGLAWHPTLWRCEWPDQV
PTCDVEAFLGFKCPAVDEYVAATNPVYPHPSDCARYFVCVEGNKPRLQVCGTRTVFDKAIGACGAPENV
PACANYYPPEPKETTY

>Daphnia magna CPAP3-D2

KGKMVADVTYCDRYWECIDGVAEQFDCPNGLVFAGRARGLLENCDYPWRGDSCEGKQLANTPISVGP
CDWKYGIFGHESSCIRYWTCWNSTATEQFCIGGLLYNEEKHACDWPEAVEGCQKHPLCKDDANGNVPL
GKSCNRYWACQGGYPRLQRCPATLVFDKRSLRCTNPPTEDCEAPPPPPPEDDPNAPEKRKERTNTSFIFV
LIILICLFFLCYLEFYLFLFYYIFKAKRKKKKKKYLYDFQLRMANQQRATQFPFRLIPNFFPSFRYTPIYLSPSVLL
AAFICEQKQKIIHFSAKFLQIDSAGTRNLKMVTGRYSPT

>TcCPAP3-A2

DPVQCDLYYVCSKGEYEEKLCPDGLVFDARDPNHERCDIPANVDCDERTELQEPHPSPGCPRANGYYRH
SDPLACDKFFNCVNGVPHELPCPPGLIYDDTASTCAWPDDSHRKDCKNAKRDKLDDGFTCPDEEILGPG
GRKLPHPTFAHPEDCGKFYICRNGVMPQKGQCVKGLVYNEETFTCDDPKNVPGCEDYYEKAEKSKTKKA

>TcCPAP3-A1

NAQFKCPPKDGQYEDPRQCDKYYECEEGVAREKLCPDGLVFDPLIRKINKCDQPFNVDCGDRTELQPPKP
NHFCPRRNGFFAHPDPAVCNKFYNCIEGEHTEITCTAGLHFDEFTGTCVWPDAAGRQGCNKDVTNKLK
DGFECPKDGQTDANGQLVVHPKYAHPTDCQRFYVCLNGQEPRDLGCQVGEVYNEESQRCDAPENVPG
CEDWYKDEPAPAKPAKKV

>ApCPAP3-A1

HLTNGQFQCPKKNGQYEDPVQCDKFYECKDGVATTKLCPDGLVFDPLNRKVNKCDQPFSVDCGERSEL
QNPQPSYLCPRRNGYFAHPDEKVCNIFYNCIEGDGTEIVCPNGLHFDEYAGSCAWPATAGRSGCNESDD
MKLKDGFTCPKDKAFNSRGQNVAHPVFAHPDDCQKFYVCLNGITPREQGCSTGEVFNEESQKCDQPEN
VAGCENWYKDDPQVQQQQQNKNKKQ

>ApCPAP3-D1

ATVIQCQEYSGSSQCPEQHGEQTYAHPDYCDQFYLCTNGTLTLEQCGNGLLYDGKGAAYHHCNYHWAV
DCGNRKAELAPISSPGCEYQFGLFSDGSACSTNYVKCEHGTPYALPCEPGLAYDDRIKKCNWPDELVDVG
CNPADIIGFSCPEKADPHSVSAKFEPYPRYALPGDSHRLITCVHGHPRLISCGEDSVVDESSLTCVEKGPHY
RKRK

>DmObstructorA

ADEVQCDKFYVCDDGVAKAKLCPDGLVFDPLNRKFNKCDQPFNVDCEDRTELQEPKSSKYCPRKNGFFA
HPDPAVCNIFYNCIEGDALETKCTVGLHFDEYSGTCVWPDTAKREGCNPEQRTSETGFVCPKDQPKTDD
RGQVVTHPKYPHPTDCQKFYVCLNGEDPRDLGCQLGEVYNDATEMCDAPENVPGCEDWYKDVDDKK
D

>DmObstructorB

GGFRGSNSQFRVGPGHPPSQRHLPPRNRDPVQEASVVPKSKQTAAEKEYEPTEECPEPNGFYPDSKQCD
KYYACLDGVPTERLCADGMVFNDYSPIEEKCDLPYNIDCMKRSKLQTPQPSLHCPRKNGYFGHEKPGICD
KFYFCVDGQFNMITCPAGLVFNPKTGICGWPDQVGVTGCKSEDVFDFECPKVNESIAVTHPRYADPNDC
QFFYVCVNGDLPRRNGCKLGQVFDEEKETCDWARKVPDCADWYKDRLTDKELDELENPKPKTTTTKRPP
RVRGQSRRKPQPKRVEPEQVKEEEEEEEE

>DmObstructorJ

CRISDPWQMLPHKDHCQRFYVCTGDDDMPFQEFNCPAEYHFSKKLMICVPGACTDESVFCGLTNSVER
VQSDCTRYRQCLEGGSFAVAKCSVGNYFDPARRACLPVAISAAHQCSCVLPDNATLANPSDCETYFRCHS
GQAELVQCPSGDYFDERVSSCVPDHTGICLEKPTMPPTLTEQALAMDECIRTGSRLAPHSRDCQRYYICA
KKRVLEMRCPRGQYFDVVRRYCALDLGSECQALQAEKQDLELEENIQVELEKNIQMDIPAKHKVKNQGH
KPVQKNSEKEVVRDQELPVPQANDKLPQPSAEAVSSYDKFSSFISM

>DmObstructorF

KVRHTVGHLSHICLGRQEGDLVPHPLDCNGYFSCSRVPTLLYCDQGLQFDENRAICDLPENTNCRPVATG
TVESANGLADNSELNWWPHKPKPVFVAVDVTSGQPVNPMEKYDPEHIECRHYGAYFLPHPRNCGLYFI
CAYGHLHRHQCGRGTAWNFEKSECQLSDQAICYGESQISEPHTDVETTMKVPTANSEGAVTVCYIVGSS
EYTTLQQFLTSPEITELPPVTPPSPPRAEANALTCPSTKQSYMSHPEDCSKYYICIGGMPVLTSCPKGLFWD
QKSGFCEMEKNVKCFQK

>Daphnia magna CPAP3-A

TQKFQCPDHKNKQYEDEVQCDKYYDCVDGVAREKLCPDGLVFDASIRRVNKCDQPFNVDCGDRTELQP
PRGTNEYCPRKNGFFAHPDSTICDLFYQCVDGEYVENRCAAGLHFDEYSGTCVWPATANREGCTENEKK
LKDGFQCPNDKNKNDANGQIVAHPHYAHPEDCQKFYVCLNGIEPRELGCAAGEVFNDDIKKCDSPDNV
PGCEDWYKDADKN

>Daphnia magna CPAP3-B

GQSNAXXXLLWQPTRLASPGQEQVPVLGLKTVQSTLKGKSHRPPMPLLAPRSAKTKPSFRTATWIIVSSIY
LLLVFGDTHTDTQTKQLHKMAKYFIAFLLVVSSVYGQQPESDCPEKNGVFADSLQCDRYYECENFVLSEKL
CDDGLVFADLGANSGIGGRCDFPFNVDCTGRAELQPANATANCPRQNGYFAHSDPTICNQFFFCSSGQ
ANLITCPDGLVFNPNTGTCSWPGEANRVGCQSKDVVAFDCPARVLEAEPTGPQFIAPLYADPTDCQYFY
VCIGGKEPRRNGCTTGLVFNDLTKRCDRPRNVPDCVDWYKTSEGDLLEPDVLEEEIVPAPKRTNIAGRS

>DpCPAP3-B1

GRDTGVARQSSPYQDLSSCPEDYGLQTYAHPKNCDQFYKCANGTLTLETCENGLLFDGAGSVHNFCNYH
WATNCGDRLFELTPDPARNPESVCEYSFGLFKPTAADCDIFYYRCAYGEAEEVACDKGLAYDDRSHSCN
WPDLLLDIGCDPEKVVGFRCPDVSSLPPNSLVRQFLPFPRYAVPNDCGRLVTCVNDYPRLISCGYGSAFNE
DTLTCDDAENVPQCANYYKE

>DpCPAP3-A1

AQAFVCPPKNGQYPDPIQCDKYYICQDGVASARLCEDGLVFDSFKRSSHKCDHMHNVDCEDRTELQPP
QGNAECPRRNGIFENADPSQCHKFVDCIDGQPKHNVCPPGLHFNDASGVCTWEAAAGRTGCVREEFLE
DGFTCPKLTAAESLTEPHPRYPHPTDCQKFYVCLNGVTPREQNCDLGEVFNTNSKQCDLPENVAECIDW
YKDHPSFVPGSVTPKSSTRGNSGQK

>DpCPAP3-B2

GQQADSDCPEKNGVFADTVQCDRYYECENFVLSEKLCADGLVFADLGVNSGVGGRCDFPFNVDCKDRP
ELQPANATANCPRQNGYFAHSDPTVCDQFFFCSSGQANLITCPGGLVFNPNTGTCSWPGEANRAGCQS
KDVVAFDCPARVLEADPVGPQFIDPLYADPTDCQYFYVCIGGKEPRRNGCTTGLVFNDLTKRCDRPRNVP
DCVDWYKTSEGDLLEPDVLDEVAAPPPKRTNFAGRS

>DpCPAP3-A2

GAALPTPRCELSLRPYMMEDDEFCDKYYVCLNGTATEEFCDDGLVFDTTKDKCELPHAVQCGDRKKQQ
NPRPTSNCPRRNGMFPVKGSCDKFYHCTDGQHTLIACPPGVIFEPLVGACVHADQTNRPNCSASQVLNF
VCPHIGSGANPSASLRFGDHDRLAHPTSCRHFYMCLLTGMPRLGGCTYGLVFNPVSGRCDQPQNVRGC
EKWYGEDDPIEEDGIPDATVAPALSSRRINNSAVRTAAPAVSVARVEPGEVAKLKAHQPMRRLSTGTNRF
GLVQEDPIDSDKSDLDEE

>DpCPAP3-A3

ILAYVTNGDPVRLCRKRRFPYYIADSDQCDLFYKCKDGQITEELCPDGQVYEPESQACFMIQRVKCGRRKR
LQSPQGNALCPRLYGRFPIANECFAYSECEQGTPTKVNCPPGLIFDIDQAVCEFPDMANRTECSAEKILDF
TCPHGSNVQSDEVIPSRNSITKFNNKKQKGDCRHFFMCLKSGRPRLGGCPLGTIYNPATFFCDKPENVPE
W

>DmObstructor-H

RINADHFDECDGMDDGAFVQSWESCQSYVYCEGEESLKGDCEDGEYFDSEAGTCDIAANVSCFLDEVDE
PSDPEPETDEEEEEIPATPRPTEPPIVETPTEVDIINIAPVVRPNCPISDDPGQVIFMASNNSCTNYYLCYHG
HAMEMHCDNELYFNSLTGQCDYPDKVQCAFEDPRSHKCLPHMTEFFPHPDNCNYFYYCIKGFLTLQQC
PFYYGWDIERRSCVQIGVAKCYGNSRRIGRKAPLPPRKQLIKS

>DmObstructor-G

LAVVQNGFAFKTSLCEGKNGGLLPMFGSCKGYYVCADGNAVTGTCEKNTLFNPLTLHCDDPDNVDCIFD
GKDNIVDDTSSSESDEDDDEEMAKTDPPVTVKATKKPRPTTLDKMCAGKKDGVMLTKNGSCQEYYVCK
AKKPHLRSCPDKQHFSPTRRICMKASEAKCSGGTRENKESDGPATTGGVCSDEKENSLVAHRSDCGKFM
LCSNMMFLVMDCPTGLHFNIATSRCDYPKIAKCQTKLNESKSKSKKPVRKSKKRRF

>DmObstructor-I

QTGKGLGNGSYLELRLDAAAESCPDDYYFNETIQACVSTDTTSCTNHQIGKCPMATEMDEFCVCKDKHL
QIWKCPEGTYFDANRLVCRVGSVECQDDYTPSPCPNSTASDVFCLCIDGKWHLNYCPTGFTFDDELQICL
NTGSDDDELPSSSGKCQRLGLFGDPADCSGYYHCREKGSDIEYFRCSVGTIFNLISFACVTGTC

Gel blots pictures

Columns key of RT-PCR gel blots from left to right

1. Nc
2. molar-forming epithelium - Intermolt
3. carapace cuticle-forming epithelium - Intermolt
4. gastrolith-forming epithelium - Intermolt
5. hepatopancreas - Intermolt
6. abdominal muscle – Intermolt
7. molar-forming epithelium – Early premolt
8. carapace cuticle-forming epithelium - Early premolt
9. gastrolith-forming epithelium - Early premolt
10. hepatopancreas - Early premolt
11. abdominal muscle – Early premolt
12. molar-forming epithelium - Late premolt
13. carapace cuticle-forming epithelium - Late premolt
14. gastrolith-forming epithelium - Late premolt
15. hepatopancreas - Late premolt
16. abdominal muscle – Late premolt
17. molar-forming epithelium - Postmolt
18. carapace cuticle-forming epithelium - Postmolt
19. gastrolith-forming epithelium - Postmolt
20. hepatopancreas - Postmolt
21. abdominal muscle – Postmolt

CqCPAP3A transcript blot

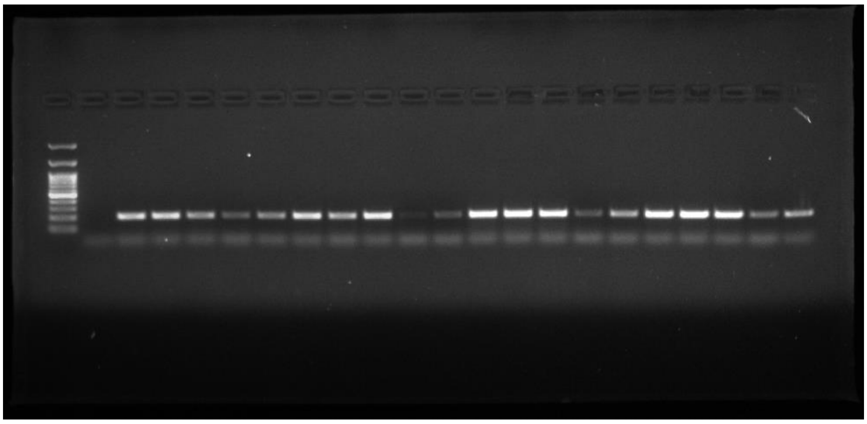

CqCPAP3E transcript blot

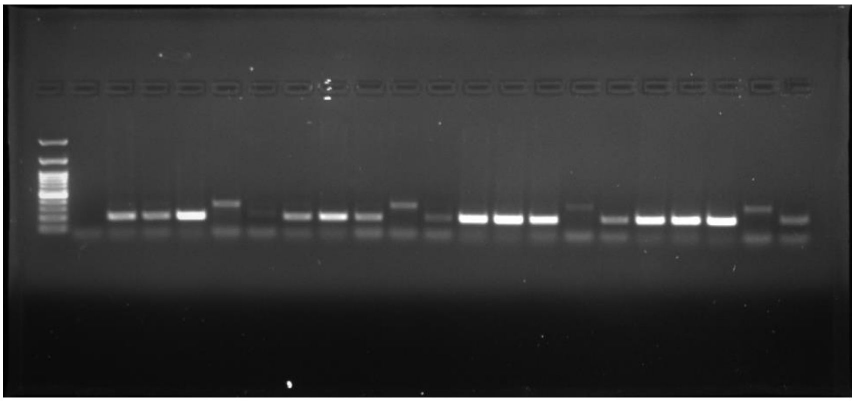

CqCPAP3B1 transcript blot

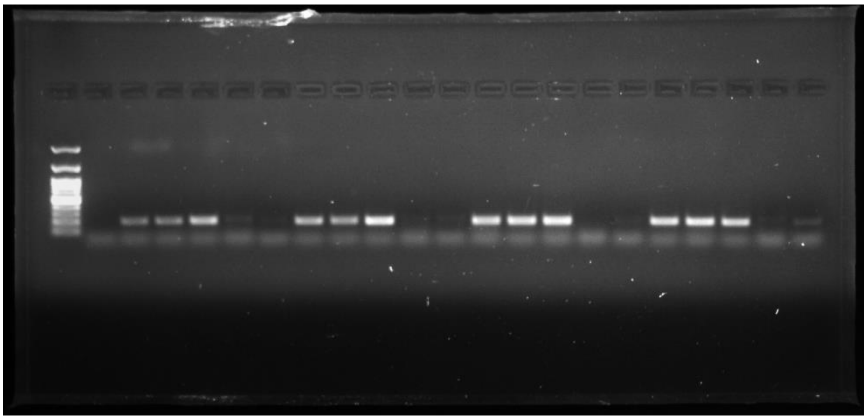

CqCPAP3B2 transcript blot

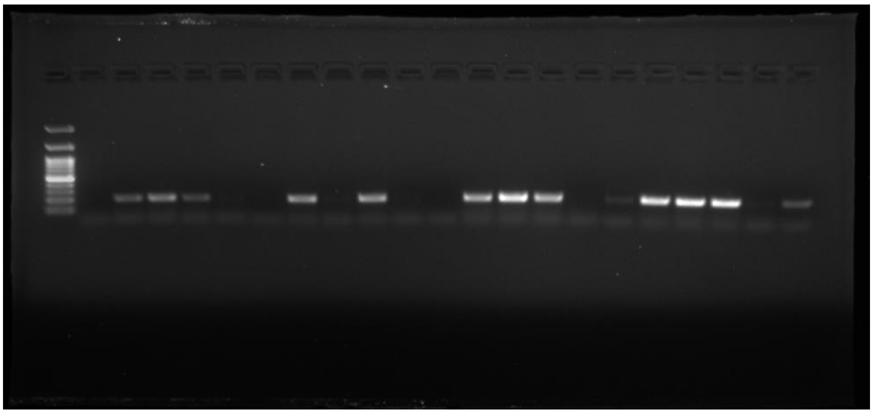

CqCPAP3I transcript blot

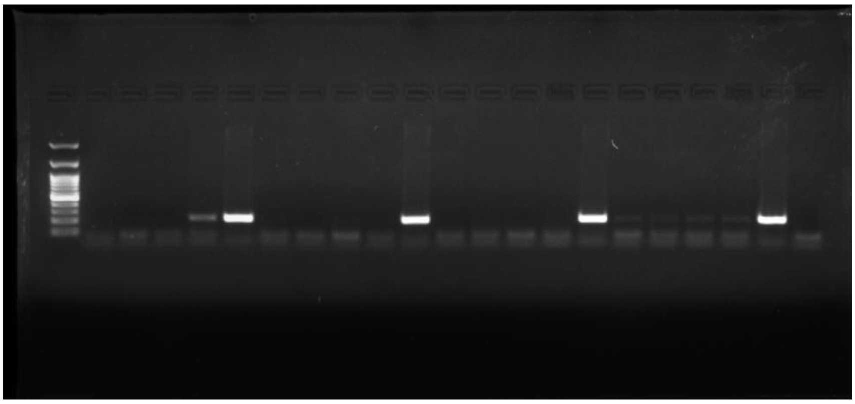

CqCPAP3J1 transcript blot

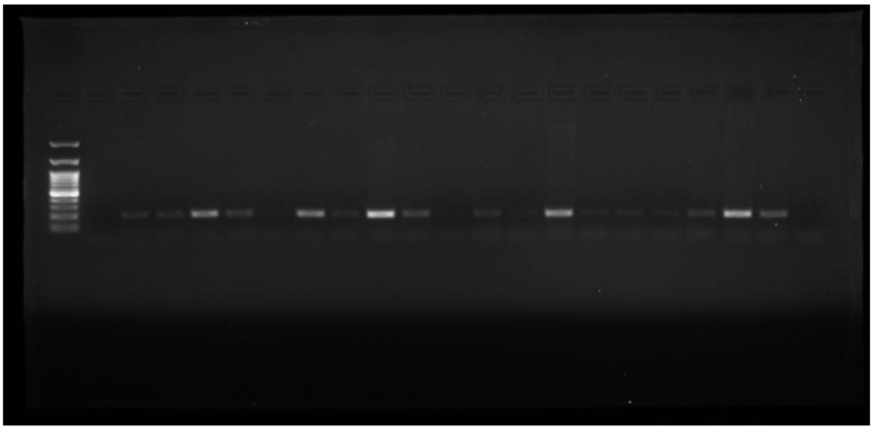

CqCPAP3J2 transcript blot

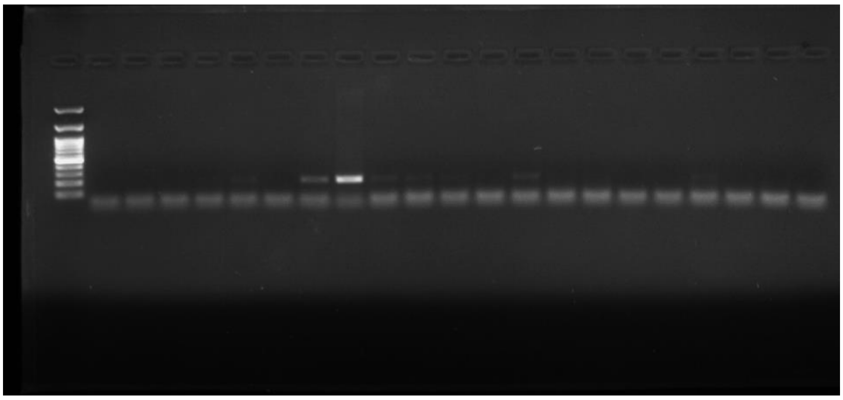

Protein Blot

First extraction

Columns key of gel blot of first extraction from left to right

1. Cuticle EGTA
2. Cuticle EGTA
3. Cuticle EGTA
4. Cuticle Urea
5. Cuticle Urea
6. Cuticle Urea
7. Gastrolith EGTA
8. Gastrolith EGTA
9. Gastrolith EGTA
10. Gastrolith Urea
11. Gastrolith Urea
12. Gastrolith Urea
13. Molar Urea
14. Molar Urea

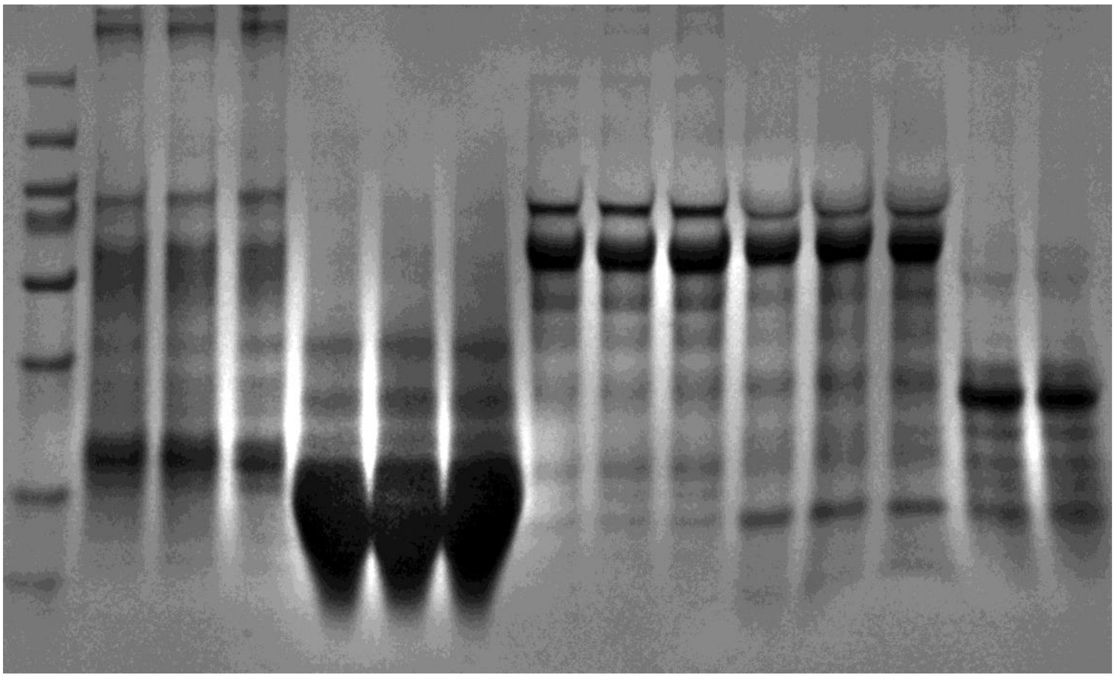

Second extraction

Columns key of gel blot of second extraction from left to right

1. Cuticle guanidinium
2. Molar Urea

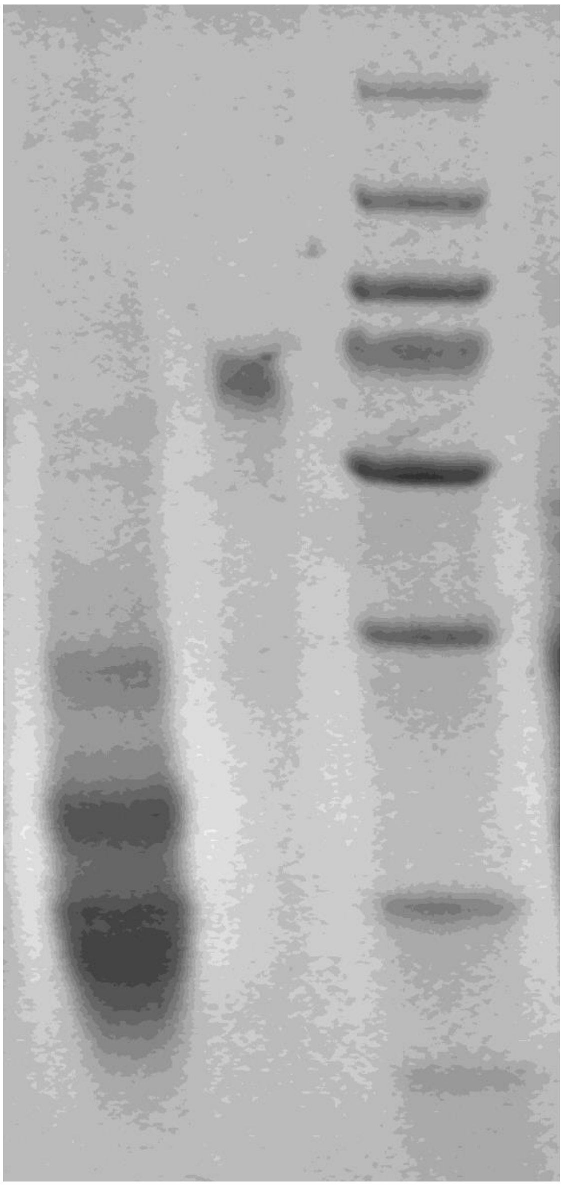

Supplement: Supplementary file 1 — Supplemental information [file 41598_2018_20835_MOESM1_ESM.pdf]
